# Specialized Pro-Resolving Lipid Mediators and Dietary Omega-3/6 Fatty Acids in Selected Inflammatory Skin Diseases: A Systematic Review

**DOI:** 10.3390/antiox15010009

**Published:** 2025-12-21

**Authors:** Angelika Biełach-Bazyluk, Olivia Jakubowicz-Zalewska, Hanna Myśliwiec, Iwona Flisiak

**Affiliations:** Department of Dermatology and Venereology, Medical University of Bialystok, 15-540 Bialystok, Polandhanna.mysliwiec@umb.edu.pl (H.M.);

**Keywords:** specialized pro-resolving lipid mediators, fatty acids, omega-3, psoriasis, atopic dermatitis, acne vulgaris, hidradenitis suppurativa

## Abstract

Specialized pro-resolving mediators (SPMs), including lipoxins, resolvins, protectins, and maresins, actively terminate inflammation and restore tissue homeostasis. This review addresses how specialized pro-resolving mediators (SPMs) and their omega-3/omega-6 PUFA precursors influence inflammatory pathways, disease mechanisms, and therapeutic potential across major inflammatory skin disorders. MEDLINE/PubMed was searched on 4 October 2025. Eligible studies included experimental, animal, mechanistic human, and interventional research examining SPMs or omega-3/omega-6 fatty acids. Non-English articles, reviews, conference abstracts, and dietary questionnaire–only studies were excluded. Two reviewers independently screened and extracted data. Due to heterogeneity, a narrative synthesis was performed. No formal risk-of-bias assessment was undertaken Of 359 records, 57 studies were included (26 psoriasis, 24 atopic dermatitis, 7 acne; scarce hidradenitis suppurativa data). Preclinical data consistently demonstrated that SPMs modulate key inflammatory pathways, support epithelial repair, and help restore immune balance. Human studies revealed altered cutaneous and systemic lipid mediator profiles—characterized by reduced omega-3–derived SPMs and predominance of omega-6-driven inflammatory mediators—suggesting impaired resolution mechanisms across these disorders. Interventional studies showed that omega-3 supplementation may reduce inflammatory markers, improve barrier function, and alleviate clinical symptoms. Early evidence on SPMs analogues and receptor agonists indicates promising therapeutic potential, but clinical data remain sparse. The body of evidence is limited by scarce human data, small sample sizes, heterogeneous interventions and variable methods. Many studies rely on subjective or non-standardized clinical outcomes, and the predominance of experimental models further limits the translational relevance of current findings. In summary, disturbances in PUFA-derived lipid mediator pathways and insufficient activation of pro-resolving mechanisms may contribute to the persistence of cutaneous inflammation. Omega-3 supplementation and SPMs-based novel therapies therefore represent plausible adjunctive approaches; however, their therapeutic relevance requires confirmation in future mechanistic and clinical studies.

## 1. Introduction

Historically, the resolution of inflammation was considered a passive process that occurred naturally once pro-inflammatory signals diminished. This long-standing concept was overturned by discoveries at the end of the 20th century, which revealed that inflammation resolution is an active and tightly regulated process initiated within the earliest hours of the inflammatory response [1]. Resolution begins with a granulocyte-initiated biochemical switch that redirects arachidonic acid (AA) metabolism from the production of pro-inflammatory prostaglandins and leukotrienes toward the generation of lipoxins. Lipoxins, together with resolvins, protectins, and maresins, constitute a class of specialized lipid mediators known as specialized pro-resolving mediators (SPMs) [2]. These bioactive lipids are enzymatically synthesized from dietary omega-3 and omega-6 PUFAs, including eicosapentaenoic acid (EPA), docosahexaenoic acid (DHA), and AA [2].

SPMs play a pivotal role in concluding inflammatory responses by restricting neutrophil migration [3], promoting macrophage-mediated clearance of apoptotic cells and debris (efferocytosis) [4], and supporting tissue repair and regeneration—all without impairing host immune defense [5]. SPMs limit neutrophil recruitment by reducing chemotaxis, impairing adhesion to endothelial cells, and restricting migration across vascular and epithelial barriers [6]. Moreover, SPMs consistently boost macrophage efferocytic capacity, as mediators such as resolvin D1 (RvD1) and its aspirin-triggered epimer were found to enhance clearance of apoptotic neutrophils [7], while maresin 1 (MaR1) markedly strengthens both efferocytosis and antibacterial phagocytosis [8]. This improved clearance further amplifies local SPMs generation, reinforcing pro-resolving pathways [9]. SPMs redirect macrophages from pro-inflammatory toward pro-resolving states by promoting reparative gene programs and suppressing nuclear factor kappa-light-chain-enhancer of activated B cells (NF-κB)–driven cytokine production [10]. Growing evidence shows that SPMs modulate macrophage metabolism by promoting oxidative phosphorylation and fatty-acid oxidation, as well as enhancing mitochondrial function, thereby favoring reparative, pro-resolving functions over pro-inflammatory activity [11]. Beyond their established effects on innate immunity, SPMs also influence adaptive immune responses by limiting memory B-cell proliferation and supporting regulatory T-cell activity [12,13]. They can promote Treg differentiation from naive T cells, enhance Treg functional properties, and indirectly favor Treg-mediated suppression of pro-inflammatory T-cell subsets, thereby contributing to the resolution of inflammation and maintenance of immune homeostasis [12,14]. Inadequate production or dysregulation of these mediators can result in incomplete resolution, contributing to the persistence and progression of various chronic inflammatory and autoimmune conditions [5].

Chronic inflammatory skin disorders, including psoriasis, atopic dermatitis (AD), acne vulgaris, and hidradenitis suppurativa (HS), share overlapping pathogenic mechanisms. These include dysregulation of both innate and adaptive immune responses [15,16,17,18], cytokine-driven inflammation [15,18,19,20], excessive keratinocyte activation [21,22,23], and disruption of skin barrier integrity [18,24]. Despite advances in treatment—such as biologic therapies targeting specific cytokines—current strategies largely focus on suppressing inflammation rather than restoring immune balance and physiological resolution. Consequently, there remains a need for approaches that actively promote the natural termination of inflammation while minimizing long-term immunosuppression.

SPMs offer a novel avenue in this regard, as they restore tissue homeostasis without directly inhibiting immune activation. Their biosynthesis from dietary omega-3 and omega-6 fatty acids (FAs) further suggests that nutritional interventions could serve as practical adjuncts for modulating chronic inflammation. Additionally, SPMs levels and their metabolic pathways hold promise as biomarkers for disease activity and therapeutic response, paving the way for more personalized treatment strategies in dermatology.

The present systematic review aims to provide a comprehensive overview of the evidence regarding SPMs and their omega-3/omega-6 FAs precursors in selected inflammatory skin diseases, including psoriasis, AD, acne vulgaris, and HS. Specifically, this work aims to (i) summarize current knowledge on the roles of SPMs in cutaneous inflammation and resolution, (ii) evaluate interventional studies investigating omega-3/omega-6 FAs supplementation, and (iii) explore the translational potential for therapeutic development and biomarker discovery.

## 2. Materials and Methods

This systematic review was conducted in accordance with the PRISMA 2020 guidelines. To promote transparency, the full study protocol and search strategy have been archived and are publicly available at https://doi.org/10.17605/OSF.IO/FV9PR. Following peer-review suggestions, the protocol was amended to include a narrative synthesis of the data and an assessment of risk of bias for studies included in the synthesis. We systematically searched MEDLINE (via PubMed) on 4 October 2025, using Medical Subject Headings (MeSH) without date restrictions. The objective of this review was to evaluate the available evidence on SPMs and their dietary precursors—omega-3 and omega-6 FAs—with a focus on their role in the pathogenesis and therapeutic potential of inflammatory skin diseases, including psoriasis, AD, acne vulgaris, and HS.

The PubMed search strategy combined terms for inflammatory skin diseases (“Psoriasis”, “Dermatitis, Atopic”, “Acne Vulgaris”, “Hidradenitis Suppurativa”) with terms related to lipid mediators and fatty acids (“Lipoxins”, “Docosahexaenoic Acids”, “Eicosapentaenoic Acid”, “Fatty Acids, Omega-3”, “Fatty Acids, Omega-6”) using the Boolean operator AND. The complete PubMed search string is provided in the Supplementary Material.

All retrieved records were merged, and duplicates were removed. Eligible studies included original experimental, animal, and human studies addressing the role of SPMs, or omega-3/omega-6 FAs in the context of inflammatory skin diseases. Non-English publications, conference abstracts without full text, narrative reviews, editorials, and commentaries were excluded from the analysis. Interventional studies involving omega-fatty acid supplementation as well as observational studies based on objective biochemical measurements of fatty acid profiles were included, while studies relying solely on self-reported dietary intake were excluded.

To achieve a comprehensive understanding of the SPMs system in inflammatory skin diseases, this review integrates evidence from two main streams: (i) studies measuring or directly reporting SPMs (lipoxins, resolvins, protectins, maresins) in humans, animal models, or experimental systems, and (ii) interventional studies involving the administration of long-chain omega-3 FAs (EPA, DHA) on the course of the disease. The collected evidence was synthesized and compared across these two streams using narrative approach to highlight consistent patterns and mechanistic insights.

Titles and abstracts of all retrieved records were independently screened by two reviewers (A.B.-B. and O.J.-Z.), with disagreements resolved by a third reviewer (H.M.). Full-text articles of potentially relevant studies were then assessed for eligibility. Data extraction was performed independently by two reviewers (A.B.-B. and O.J.-Z.) using a standardized form. Extracted information included study design, population, intervention/exposure characteristics, analytical methods, lipid mediator measurements, and clinical outcomes. We extracted all reported outcomes related to SPMs concentrations, omega-3/omega-6 fatty acid profiles, clinical disease severity, and mechanistic inflammatory markers, irrespective of measurement methods or time points. Additional variables included participant characteristics, disease subtype, intervention dose and duration, model system, and analytical methods; missing or unclear information was extracted as reported without assumptions. Due to heterogeneity in study designs, analytical methods, and outcome measures, the results were summarized using a structured narrative synthesis framework, and therefore no effect measures were predefined, nor were reporting bias or certainty assessments performed.

## 3. Results

The search resulted in the retrieval of 359 records of which 354 were screened for relevance. Seventy-seven full-text articles were assessed for eligibility, and 20 were excluded. The reasons for exclusion were as follows: (1) assessment of omega-3 exposure based exclusively on food frequency questionnaires (*n* = 6) [25,26,27,28,29,30], nutritional counseling (*n* = 2) [31,32], or multi-ingredient supplements not specific to omega-3 fatty acids (*n* = 1) [33]; (2) outcomes unrelated to inflammatory skin diseases (*n* = 6) [34,35,36,37,38,39]; (4) ineligible study design, including observational studies focused solely on metabolic biomarkers (*n* = 2) [40,41]; and case report series (*n* = 1) [42]; and (5) insufficient or missing data on pro-resolving or anti-inflammatory markers (*n* = 2) [43,44]. Fifty-seven studies were ultimately included in qualitative synthesis.

**Figure 1:**
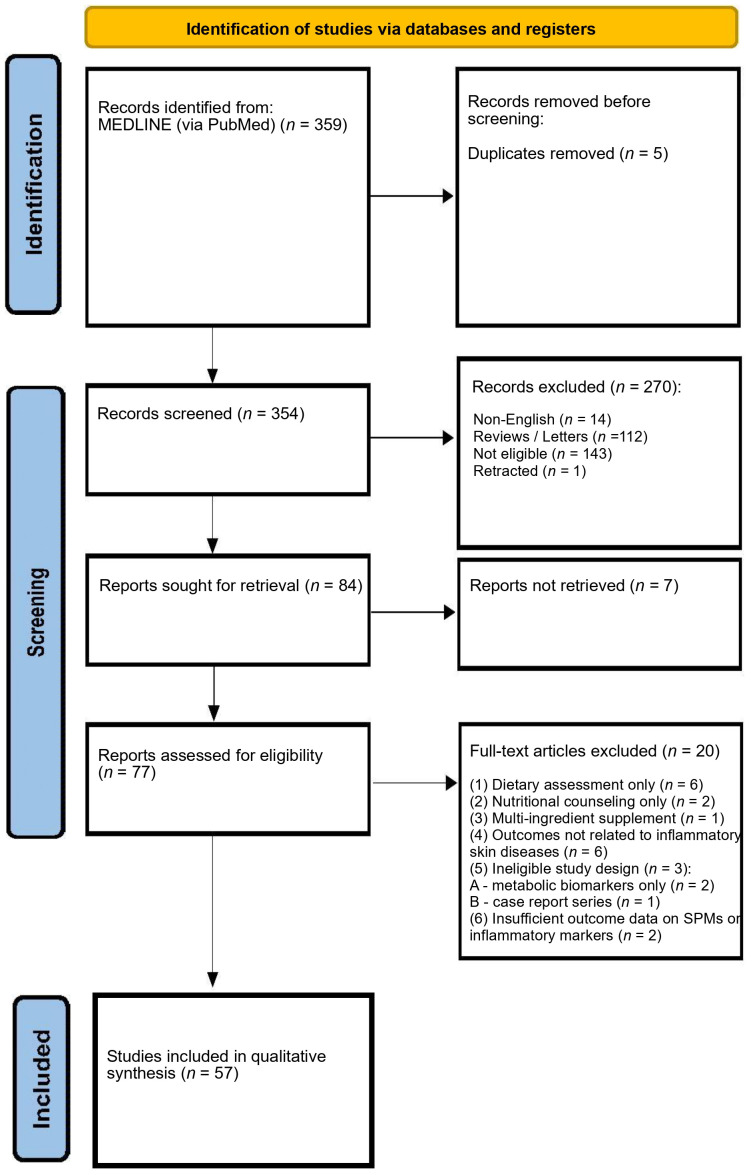
PRISMA 2020 flow diagram illustrating the study identification, screening, eligibility assessment, and inclusion process.

The flowchart presenting full search strategy is demonstrated in Figure 1.

The basic characteristics of the original papers on the researched topic are summarized in Table 1, Table 2, Table 3 and Table 4. Each table includes study design, population characteristics, intervention or exposure details, biochemical endpoints related to omega-3 fatty acids or SPMs, and clinical outcomes relevant to inflammatory skin diseases. In total, 21 experimental, 28 human interventional, and 8 human observational studies were identified, including two that combined in vitro and human investigations. Of the 57 studies, the majority focused on psoriasis (*n* = 26) and atopic dermatitis (*n* = 24), with a smaller number addressing acne (*n* = 7). Given the heterogeneity of included studies, which range from in vitro and animal models to mechanistic human investigations and clinical interventions and the focus on mechanistic insights into SPMs and their precursors, a formal risk of bias assessment was not conducted. Results of individual studies are presented narratively in the disease-specific sections and corresponding tables; quantitative effect estimates were inconsistently reported and could not be standardized. No meta-analysis was conducted, and consequently, heterogeneity analyses, sensitivity analyses, reporting bias assessments, and certainty-of-evidence evaluations were not undertaken.

**Table 1:** Effects of Specialized Pro-Resolving Lipid Mediators on Psoriasis Pathophysiology.

Author	Year	Population	Design/Intervention	Comparator	Key Observation
		Experimental and Preclinical Studies			
Chen et al. [45]	2011	NHDF and NHEK	Treatment with lipoxin A4	Untreated control cells	LXA4 suppressed the expression of IL-6 and IL-8 in keratinocytes and fibroblasts.
Liu et al. [46]	2017	IMQ-induced psoriasiform dermatitis mice model LPS-induced keratinocytes	Treatment with BML111 (LXA4 receptor agonist)	IMQ-induced mice without treatment (vehicle control)	BML111, a lipoxin A4 receptor agonist, treatment reduced IMQ-induced psoriasiform dermatitis. The effect of BML111 and lipoxin A4 is mediated by through HMGB and subsequent downregulation of inflammatory pathways.
Xu et al. [47]	2018	IMQ-induced psoriasiform dermatitis mice model	Pretreatment with RvD1	IMQ-induced mice without treatment (vehicle control)	Pretreatment with RvD1 mitigates IMQ-induced psoriasiform dermatitis by targeting ALX/FPR2, and consequent inhibition of the IL-23/IL-17 axis.
Sawada et al. [48]	2018	IMQ-induced psoriasisiform dermatitis mice model	Treatment with RvE1	Untreated or IMQ-induced control mice	RvE1 markedly reduced inflammatory infiltration, epidermal hyperplasia, and IL-23 expression in psoriatic skin. RvE1 inhibited IL-23 production and migration of dendritic cells and IL-17–producing γδ T cells through BLT1 antagonism.
Park et al. [49]	2021	IMQ-induced psoriasiform dermatitis mice model NHEK	Treatment with PD1	Untreated or IMQ-induced control	PD1 mitigated psoriatic symptoms, epidermal thickening, erythema, and scaling. PD1 reduced pro-inflammatory cytokines and suppressed STAT1 and NF-κB activation in skin lesions. PD1 alleviated systemic inflammation and cytokine expression in keratinocytes.
Sorokin et al. [50]	2023	Mouse model of psoriasis-like inflammation	Dietary supplementation with DHA or EPA	Normal diet control	DHA supplementation increases skin levels of RvD5, protectin DX, and maresin 2.EPA supplementation reduces skin PGE_2_ and TXB_2_ levels.
		Observational and Mechanistic Human Studies			
Sorokin et al. [51]	2018	Psoriatic patients	Psoriatic patients compared with healthy controls: blood samples, lesional and nonlesional skin punch biopsies.	Healthy controls (matched blood and skin samples)	14-HDHA, 17-HDHA, precursors of D-series resolvins and protectins) are elevated in psoriatic skin lesions RvD2 is detected in ~50% of psoriasis and healthy individuals (no significant quantitative difference reported) 14,15-EpETE; 17,18-EpETE are mostly undetectable in both psoriasis and control groupsA shift toward ω-6 pro-inflammatory dominance and relative deficiency of ω-3-derived pro-resolving mediators in psoriasis
Sorokin et al. [52]	2018	Psoriatic patients (blood samples, lesional and nonlesional skin punch biopsies.) NHEK	NHEK cultivated in complete keratinocyte growth media-2 and subsequently treated with vehicle or TNFα or RvD1/RvD5 or TNFα, RvD1 and RvD5.	Vehicle-treated or TNFα-only treated NHEK	RvD5, protectin DX, and lipoxin are found only in lesional PSO skin, while protectin D1 is present in nonlesional PSO skin. RvD1 and RvD5 decrease IL-24 and S100A12 expression in human keratinocytes.

Abbreviations: ALX/FPR2, lipoxin A4/formyl peptide receptor 2; BLT1, leukotriene B4 receptor 1; DHA, docosahexaenoic acid; EPA, eicosapentaenoic acid; HMGB, high-mobility group box protein; IL, interleukin; IMQ, imiquimod; LPS, lipopolysaccharide; LXA4, lipoxin A4; NF-κB, nuclear factor kappa-light-chain-enhancer of activated B cells; NHDF, normal human dermal fibroblasts; NHEK, normal human epidermal keratinocytes; PD1, protectin D1; PGE_2_, prostaglandin E2; PSO, psoriasis; RvD1, resolvin D1; RvD2, resolvin D2; RvD5, resolvin D5; RvE1, resolvin E1; SPM, specialized pro-resolving mediator; STAT1, signal transducer and activator of transcription 1; TNFα, tumor necrosis factor alpha; TXB_2_, thromboxane B2.

**Table 2:** Clinical and Preclinical Evidence of ω-3 Polyunsaturated Fatty Acids in Psoriasis.

Author	Year	Population	Design/Intervention	Comparator	Key Observation
Experimental and Preclinical Studies					
Karrys et al. [53]	2018	Human keratinocytes (HEKn) homozygous for LCE3C_LCE3B deletion	In vitro DHA and curcumin treatment (VDR ligands)	Untreated/TNFα-stimulated cells	DHA and curcumin increased LCE3A/D/E expression, reduced MAPK activation, suggesting anti-inflammatory and skin barrier–repair effects relevant to psoriasis
Wannick et al. [54]	2018	Mouse model of Aldara-induced psoriasis-like dermatitis	Preclinical study; treatment with Compound A, a potent synthetic FFA4/GPR120 agonist	Vehicle-treated mice	Compound A did not improve psoriasis-like dermatitis, suggesting that sole FFA4 activation is insufficient to mediate the anti-inflammatory effects of ω3-PUFAs
Morin et al. [55]	2021	In vitro psoriatic and healthy skin substitutes	10 μM DHA supplementation in culture medium	Unsupplemented control media	DHA reduced abnormal keratinocyte differentiation, decreased PGE_2_ and 12-HETE, rebalanced PPAR expression, and lowered TNF-α, attenuating psoriatic features.
Simard et al. [56]	2021	reconstructed 3D skin substitutes: psoriatic and healthy	In vitro 3D skin models derived from healthy and psoriatic donor cells, with keratinocytes cultured in 10 μM α-linolenic acid (ALA, ω-3 PUFA) medium	Skin substitutes cultured in standard medium (ALA)	ALA reduced keratinocyte proliferation (↓ Ki67), improved differentiation (↑ filaggrin, loricrin), incorporated into epidermal phospholipids, metabolized to EPA and ω-3 DPA, increased 15-HEPE and 18-HEPE, decreased ω-6 lipid mediators, and activated ERK1/2 signaling, normalizing psoriatic phenotype
Son et al. [57]	2022	FFA4 WT and KO BALB/c mice with imiquimod-induced psoriasis-like skin lesions	In vivo animal study; treatment with Compound A (30 mg/kg), a selective FFA4 agonist	Vehicle-treated mice (imiquimod only); FFA4 KO mice	Compound A reduced PASI, epidermal thickness, keratinocyte proliferation, TH17/TH1 cytokines, and CD4^+^IL-17A^+^ T cells in FFA4 WT mice; no effect in KO mice, indicating FFA4-mediated anti-psoriatic ω-3 PUFA effects.
Morin et al. [58]	2023	In vitro healthy and psoriatic skin substitutes with polarized T cells	Culture media supplemented with 10 μM EPA	Unsupplemented psoriatic skin model	EPA increased epidermal EPA, DPA, and DHA, elevated anti-inflammatory lipid mediators (PGE_3_, 12-HEPE, EPEA), and reduced ω-6–derived metabolites, restoring lipid homeostasis.
ω-3 Fatty Acids Intervention Studies on the Course of Psoriasis					
Gupta et al. [59]	1989	18 patients with stable plaque psoriasis treated with UVB	15-week RCT; diet enriched in fish oil (3.6 g of EPA 2.4 g of DCHA)	diet enriched in olive oil (76% olecic acid, 9% palmitic acid, 7% linoleic acid, 2%, stearic acid).	Fish oil supplementation led to greater improvement in BSA, redness, scaling, erythema, epidermal thickness, and overall global response compared to olive oil diet.
Dewsbury et al. [60]	1989	11 psoriatic patients treated simultaneously with topical EPA and vehicle creams on different lesions	7-week single-blind within-patient comparison; topical EPA	placebo (vehicle) cream	8/11 patients showed subjective and objective improvement with EPA-treated plaques vs. placebo
Kojima et al. [61]	1991	9 patients with chronic stable psoriasis	EPA supplementation 3.6 g/day for up to 12 months	Baseline/pre-treatment status	Modest but sustained clinical improvement; increased plasma EPA and LTB_5_, reduced LTB_4_
Grimminger et al. [62]	1993	21 patients with guttate psoriasis	Double-blind RCT;IV ω-3 emulsion (4.2 g EPA+DHA daily for 10 days)	IV ω-6 lipid emulsion	*ω-3* group showed rapid, significant clinical improvement and increased EPA-derived mediators; ω-6 group showed only slight changes
Søyland et al. [63]	1993	124 adults with stable plaque psoriasis	4-month, double-blind RCT; six capsules daily of highly concentrated fish-oil ethyl esters (51% EPA, 32% DHA)	Six capsules daily of corn oil (oleic acid 18:1 n-9, linoleic acid 18:2 ω-6)	Fish-oil raised serum ω-3 PUFA but showed no PASI or symptom improvement vs. corn oil; minor reductions in scaling and infiltration seen in both groups.
Henneicke-von Zepelin et al. [64]	1993	52 patients with moderate to severe psoriasis	Multicenter, double-blind RCT; topical ω-3 PUFA cream (1% or 10%) applied for 8 weeks	Placebo cream applied to matched control lesion on the same patient	Both treated and placebo lesions improved vs. baseline, but no significant difference between groups. Topical omega-3 PUFA was well tolerated; one case of mild perilesional eczema.
Mayser et al. [65]	1998	83 patients with chronic plaque psoriasis	Double-blind RCT; IV ω-3 lipid emulsion (8.4 g EPA + DHA daily for 14 days)	IV ω-6 lipid emulsion	Greater PASI reduction in ω-3 group (*p* = 0.048); 37% achieved ≥50% PASI improvement vs. 23% controls; increased plasma EPA and anti-inflammatory eicosanoids
Danno et al. [66]	1998	40 patients with moderate plaque psoriasis	12-week randomized open study; combination therapy etretinate + 1800 mg/day EPA	etretinat	The combination therapy was more effective than monotherapy, especially with regard to improvement >75%
Guida et al. [67]	2014	44 obese patients with mild-to-severe plaque psoriasis on stable immunosuppressive therapy	6-month RCT; an energy-restricted diet enriched in ω-3 PUFAs (2.6 g/day) and low in ω-6 PUFAs	usual diet without nutritional intervention	ω-3 enriched diet significantly improved PASI, itch, and DLQI scores at 3 and 6 months (*p* < 0.05), and reduced body weight, waist circumference, triglycerides, total cholesterol, and ω-6/ω-3 ratio compared to controls.
Kristensen et al. [68]	2017	145 patients with psoriatic arthritis	Randomized, double-blind trial; 3 g/day marine ω-3 PUFA for 24 weeks	3 g/day olive oil	Improved disease activity measures, including PASI and reduced NSAID/paracetamol use; decreased leukotriene B4 and increased leukotriene B5 formation.
Petrovic et al. [69]	2023	64 patients with mild psoriasis	Double blind RCT; Daily oral herring roe oil capsules providing 2.6 g ω-3 PUFA (DHA:EPA 3:1 ratio)	Matching placebo capsules with coconut oil (medium-chain triglycerides)	Significant PASI reduction after 26 weeks vs. placebo. ↓ CD38^+^ CD4^+^/CD8^+^ T cells and CD56^bright NK cells, maintained monocytes, ↓ CCL2, ↑ IFN-γR1 and CXCL10. Cytokine changes correlated with clinical improvement, indicating anti-inflammatory ω-3 PUFA effects.
Huang et al. [70]	2024	Genome-wide association study datasets	Two-sample Mendelian randomization assessing the causal relationship between fatty acid levels (ω-3, ω-6, and others) and psoriasis risk.	Genetic variants associated with lower or higher fatty acid levels.	Elevated genetically predicted ω-3 fatty acids were associated with lower psoriasis risk; no causal effect observed for other FA types.

Abbreviations: ALA, α-linolenic acid; BALB/c, Bagg albino laboratory-bred mouse strain c; BSA, body surface area; CD, cluster of differentiation; CCL2, C-C motif chemokine ligand 2; CXCL10, C-X-C motif chemokine ligand 10; DLQI, Dermatology Life Quality Index; DHA, docosahexaenoic acid; DPA, docosapentaenoic acid; DCHA, docosahexaenoic acid (older abbreviation); EPA, eicosapentaenoic acid; EPEA, eicosapentaenoyl ethanolamide; ERK1/2, extracellular signal-regulated kinase 1/2; FA, fatty acid; FFA4, free fatty acid receptor 4 (GPR120); GPR120, G-protein-coupled receptor 120; HEKn, human epidermal keratinocytes (neonatal); HETE, hydroxyeicosatetraenoic acid; HEPE, hydroxyeicosapentaenoic acid; IFN-γR1, interferon gamma receptor 1; IL, interleukin; KO, knockout; LCE, late cornified envelope; LTB, leukotriene B; MAPK, mitogen-activated protein kinase; NK, natural killer; NSAID, non-steroidal anti-inflammatory drug; PASI, Psoriasis Area and Severity Index; PGE_2_, prostaglandin E2; PGE3, prostaglandin E3; PPAR, peroxisome proliferator-activated receptor; PUFA, polyunsaturated fatty acid; RCT, randomized controlled trial; TH1, T-helper 1 cell; TH17, T-helper 17 cell; TNFα, tumor necrosis factor alpha; UVB, ultraviolet B; VDR, vitamin D receptor; WT, wild-type; ↓, decrease; ↑, increase.

**Table 3:** Summary of Clinical, Experimental, and Observational Evidence on Omega-3 Fatty Acids and Specialized Pro-Resolving Mediators in Acne Vulgaris.

Author	Year	Population	Design/Intervention	Comparator	Key Observation
Khayef et al. [71]	2012	13 males with inflammatory acne	Supplementation of fish oil capsules daily for 12 weeks that contained a total of 930 mg EPA, 720 mg DHA, and 174 mg DPA per 3 capsules.	No control group.	8 patients reduced acne symptoms, but no significant changes in total lesion count or redness was observed.
Jung et al. [72]	2014	45 patients with mild to moderate acne	A 10-week double-blind RCT, parallel study including 45 patients divided in 2 groups: supplementing omega-3 (500 mg/day EPA and 500 mg/day DHA) or suplementing GLA 200 mg/day	A control group on regular diet.	Reduced mean-count inflammatory and non-inflammatory acne lesions in treatment groups compared to placebo group.
Aslan et al. [73]	2016	31 female patients with moderate or severe acne	Cross-sectional study measuring serum FA and inflammatory mediators	21 healthy controls	Significantly decreased EPA levels, increased AA/EPA and GLA/EPA ratios, increased serum LPL, increased sPLA2 in acne vulgaris patients
Zainab et al. [74]	2021	60 patients treating acne with oral isotretinoin	Double-blind RCT including 34 patients on isotretinoin (0.5 mg/kg) plus omega-3 (1 mg/kg)	26 patients on oral isotretinoin (0.5 mg/kg) and placebo	Omega-3 group had significantly fewer cases of cheilitis, lip dryness, and xerosis compared to placebo (*p* < 0.05).
Guertler et al. [75]	2024	60 patients with acne	16-week interventional study;Mediterranean diet + algal DHA/EPA (600–800 mg DHA, 300–400 mg EPA/day).	No control group; within-subject comparison across visits	Acne patients exhibited an EPA/DHA deficiency; supplementation significantly improved acne severity and reduced DLQI scores
Huang et al. [76]	2024	46 acne vulgaris patients taking isotretinoin; three- and six-week-old male Sprague–Dawley rats	RCT: isotretinoin ± ω-3 fatty acids (2.4 g/day for 12 weeks); evaluation by clinical assessment, biochemical markers, gut microbiota. Parallel animal study using acne-induced rats and fecal microbiota transplantation (FMT).	Isotretinoin alone vs. isotretinoin + ω-3 fatty acids; 20 healthy controls.	Combined ω-3 + isotretinoin produced greater clinical improvement, reduced TG, increased HDL, and restored gut microbiota diversity. In rats, ω-3 supplementation reduced inflammation and comedones; FMT from ω-3-treated donors replicated the benefit, confirming a gut–skin axis mechanism.
Zhang et al. [77]	2025	Data from randomized controlled trials on acne patients and genetic datasets (European ancestry) for MR analysis.	Mendelian randomization evaluating causal links between serum UFA metabolites and acne.	genetic non-exposure variants	MR analysis identified EPA and AA as protective factors and DGLA as a risk factor (Enzymes FADS1/FADS2 implicated in acne regulation).

Abbreviations: AA—Arachidonic Acid; DGLA—Dihomo-γ-Linolenic Acid; DLQI—Dermatology Life Quality Index; DPA—Docosapentaenoic Acid; DHA—Docosahexaenoic Acid; EPA—Eicosapentaenoic Acid; FA—fatty acids; FADS1/FADS2—fatty acid desaturase 1/2; FMT—Fecal Microbiota Transplantation; GLA—γ-Linolenic Acid; HDL—High-Density Lipoprotein; LPL—Lipoprotein Lipase; MR—Mendelian Randomization; RCT—Randomized Controlled Trial; sPLA2—Secretory Phospholipase A2; TG—Triglycerides; UFA—Unsaturated Fatty Acid; ω-3/ω-6—Omega-3/Omega-6 Fatty Acids.

**Table 4:** Summary of Clinical, Experimental, and Observational Evidence on Omega-3 Fatty Acids and Specialized Pro-Resolving Mediators in Atopic Dermatitis.

Author	Year	Population	Design/Intervention	Comparator	Key Observation
Experimental and Preclinical Studies					
Weise et al. [78]	2011	BALB/c mouse model of allergen-induced dermatitis	Dietary DHA + AA (24 mg/kg DHA + 48 mg/kg AA daily for 64 days)	Control diet, DHA alone, or AA alone	Oral DHA + AA reduced dermatitis severity and Ki67 expression, increased Foxp3^+^ T cells and IL-10, and suppressed keratinocyte TSLP; DHA or AA alone ineffective.
Kim et al. [79]	2012	DNFB-induced mouse AD model	Experimental study. Intraperitoneal RvE1 (100 or 200 ng/mouse/day) on days 7–13 after AD induction.	DNFB-treated (induction-only) and vehicle-treated controls	RvE1 in dose-dependent manner ameliorated DNFB-induced AD via downregulation of IgE and Th1/Th2 cytokine responses, and limiting immune cell infiltration (CD4^+^ T, CD8^+^ T, eosinophils, and mast cells)
Han et al. [80]	2015	DNCB-induced experimental ADmouse model	Experimental study. Oral DHA (100 mg/kg/day in drinking water) for 30 days; DHA-M2 macrophages transfused intravenously at days 12 and 19.	DNCB-induced AD without DHA or DHA-M2 transfusion; hydrocortisone group for comparison	DHA reduced IgE, histamine, ear thickness, epidermal thickness, and inflammatory cell infiltration. DHA increased CD4+Foxp3+ Tregs in LN and skin; suppressed Th1/Th2/Th17 cytokines; increased TGF-β, CTLA-4 DHA-M2 macrophages transferred protective effects, reduced inflammation, increased Tregs at inflammation sites.
Yoshida et al. [81]	2016	NC/Nga mice with AD-like dermatitis	DHA/EPA administered with FK506	FK506 alone	Combined DHA/EPA + FK506 reduced dermatitis severity, LTB_4_ levels, immune cell infiltration, serum IgE, and IL-13/IL-17A secretion; effects reversed by LTB_4_ injection, indicating LTB_4_-dependent mechanism.
Fujii et al. [82]	2018	HR-AD mice with diet-induced AD–like symptoms	Oral administration of EPA ethyl ester (EPA-E) at 600 or 3000 mg/10 mL/kg once daily for 15 days	Vehicle (oleic acid) and HR-AD diet alone	High-dose EPA-E improved AD-like skin symptoms EPA-E lowered TSLP and IL-4 but not IL-5 expression EPA-E administration restored the depleted covalently bound ceramides, which resulted in improved barrier function
Lee at al. [83]	2019	mouse models of AD and psoriasis	Dietary supplementation with 5% EPA for 4 weeks prior to disease induction; AD induced with *Dermatophagoides farinae* body extract ointment, psoriasis induced with 5% imiquimod cream	Control diet	EPA altered skin lipid profiles by reducing AA-derived mediators (PGE_2_, TXB_2_, LTB_4_) and increasing EPA metabolites (e.g., RvE_1_), but failed to reduce scratching or dermatitis severity in AD and psoriasis models.
Sato et al. [84]	2025	Rat hind paw edema (PAF-induced) and mouse model of AD	Topical squid phospholipids (1% or 5% ointment) extracted from *Todarodes pacificus*, rich in DHA, EPA, and AA	Vehicle (Vaseline), soybean PC (1%), DHA (1.2%)	ω-3–rich squid phospholipids (DHA/EPA) reduced PAF-induced inflammation and AD-like skin lesions in mice DHA or soybean PC alone ineffective
Observational and Mechanistic Human Studies					
Montes et al. [85]	2013	211 non-atopic mothers and their infants followed-up to 14 months	Prospective cohort assessing maternal and cord plasma FA composition during pregnancy and risk of infant atopic eczema	Non-eczema vs. eczema infants	Higher maternal and cord ω-3 LC-PUFAs (particularly DHA) associated with lower eczema risk
Mihály et al. [86]	2014	20 adult AD patients and 20 healthy controls	Cross-sectional study assessing plasma and PBMC fatty acid composition, FADS2 and SCD1 expression	Healthy control group	AD patients showed elevated FADS2 expression in PBMCs and increased FADS2-derived ω-6 PUFAs, with reduced ω-3 PUFA levels; suggesting altered desaturase activity may drive ω-6 dominance and impaired ω-3-mediated resolution pathways.
Gardner et al. [87]	2020	1131 mother–child pairs from a USA prenatal cohort (CANDLE study)	Observational prospective cohort. Maternal plasma phospholipid PUFAs measured during 2nd trimester; child AD assessed by ISAAC-based questionnaire at 4–6 years.	Comparison by maternal PUFA profiles (ω-3 PUFA, ω-6 PUFA, ω-6:ω-3 ratio, and combined EPA+DHA quartiles).	Maternal ω-6 PUFA exposure during pregnancy was positively associated with childhood AD, particularly among atopic mothers. ω-3 PUFAs showed no protective association overall, though moderate levels of EPA + DHA might reduce risk. No consistent link was observed for the ω-6:ω-3 ratio.
Mao et al. [88]	2025	268,589 UK Biobank participants (after exclusion of prevalent AD cases	Prospective cohort study examining associations between FA profiles and risk of AD over a median 14.4-year follow-up	Comparison across FA quintiles and genetic subgroups	Higher plasma ω -3 and non-DHA ω-3 reduced AD risk, while elevated ω -6/ω -3 ratio increased risk; SFA, MUFA, ω -6, and LA showed no association. ω -3 attenuated genetic risk linked to rs1692120 and rs174448, with ~9% of AD cases potentially preventable through ω -3 supplementation.
Interventional Human Studies					
Bjørneboe et al. [89]	1987	31 adults with moderate to severe AD and personal or family history of atopy	12-week double-blind RCT. Intervention: capsules containing fish oil (~1.8 g EPA and 1.2 g DHA).	Placebo (olive oil) capsules. Mild topical steroids allowed.	Significant patient-reported improvement in itch, scaling, and total score; no significant physician-rated change or steroid use difference.
Berth-Jones et al. [90]	1993	123 patients with AD	16-week double-blindRCT Active arms: (1) Evening primrose oil, (2) Evening primrose + fish oil,; allowed topical steroids/emollients as required.	Placebo capsules topical steroids/emollients as required.	No significant improvement in disease severity, percentage of skin affected, topical steroid use, or patient symptom
Watanabe et al. [91]	1999	64 patients with AD	Topical EPA/DHA ointment (1.2% DHA + 0.6% EPA, applied 2–3×/day for 4 weeks)	Placebo: hydrophilic ointment base applied to contralateral limb (*n* = 12 subset)	Significant improvement in erythema, papules, scaling, itching, and thickening; >80% improvement vs. 0–40% with placebo
Mayser et al. [92]	2002	22 hospitalized patients with moderate-to-severe AD	Double-blind RCTl daily IV infusion of ω-3 fatty acid-based lipid emulsion for 10 days	ω-6 lipid emulsion	IV ω-3 lipid infusion improved AD severity and elevated plasma/membrane EPA and EPA/AA ratio, inducing EPA-derived mediators without affecting lymphocyte function.
Dunstan et al.l. [93]	2003	98 atopic pregnant women; follow-up of their infants at high risk of allergic disease to 12 months	Double-blind RCT. Maternal supplementation with fish oil: 4 × 1 g/day capsules (total 3.7 g ω-3 PUFAs: 56% DHA, 27.7% EPA) from mid-pregnancy until delivery.	Olive oil capsules (placebo)	No significant difference in overall incidence of atopic dermatitis between groups. Infants in the fish oil group exhibited lower allergen sensitization rates and significantly reduced severity of AD.
Koch et al. [94]	2008	53 adults with atopic eczema; 44 completed (DHA *n* = 21, Control *n* = 23)	8-week double-blind RCT DHA 5.35 g + EPA 0.37 g/day	Isoenergetic control capsules containing MCT.	DHA supplementation significantly improved SCORAD score, reduced affected skin area, decreased plasma TG, increased HDL. Marked rise in ω-3 PUFA levels and reduced ω-6/ω-3 ratio. Ex vivo PBMC showed significant reduction in induced IgE production.
Furuhjelm et al. [95]	2009	145 pregnant women with family history of allergic disease; follow-up of their infants to 24 months	Double-blind, RCT. Maternal supplementation ω-3 fatty acids (1.6 g/day EPA + 1.1 g/day DHA) from gestational week 25 until delivery	Placebo: soybean oil (2.5 g/day linoleic acid + 0.28 g/day α-linolenic acid)	ω-3 supplementation in pregnancy reduced IgE-associated eczema in infants during the first 2 years. Overall eczema frequency similar, but fewer IgE-mediated cases in the ω-3 group. Eczema severity (SCORAD) did not differ between groups. Trend toward lower IgE-associated allergic disease overall, especially in children of non-allergic mothers. Higher DHA and lower AA/EPA ratios in mothers and infants were linked to reduced allergic risk.
Noakes et al. [96]	2012	123 pregnant women with low fish intake and atopic family history	RCT: 2 portions of salmon/week from 20 wks gestation to delivery (~3.45 g EPA + DHA; 28 µg vit D_3_)	Habitual low-fish diet	Increased cord plasma EPA + DHA and decreased AA; reduced CBMC IL-2, IL-4, IL-5, IL-10, TNF-α, and PGE_2_ responses; no difference in IgE, AD incidence, or severity at 6 months
Palmer et al. [97]	2012	Pregnant women with a personal or family history of allergic disease and their infants followed up to 1 year	Double-blind RCT. From 21 weeks’ gestation until delivery, mothers received fish oil capsules daily (800 mg DHA, 100 mg EPA).	Control group: vegetable oil capsules	Increased cord plasma DHA + EPA; decreased AA; reduced incidence of atopic eczema at 1 year; no difference in overall IgE-associated allergy or food allergy.
Wu et al. [98]	2013	60 infants (aged 1–12 months, mean 4.2 months) with acute/subacute facial atopic eczema diagnosed by AAD criteria.	Double-blind RCT. Topical 0.1% 15(R/S)-methyl-lipoxin A_4_ (LXA_4_) cream applied twice daily for 10 days.	Placebo cream and 0.1% mometasone furoate (Eloson^®^ Schering-Plough, Shanghai, China) cream.	LXA_4_ improved erythema and pruritus by day 3, papules/vesicles/scaling by day 5, and lichenification by day 10; effects persisted 1 week post-treatment. Efficacy comparable to mometasone (EASI, SSS), both improved IDQOL. No adverse events; relapse at day 40: LXA_4_ 63%, mometasone 53%
Komulainen et al. [99]	2023	439 pregnant women (BMI ≥ 25 kg/m^2^, <18 weeks gestation, no chronic disease) and 287 infants followed to 24 months	Double-blind RCT. Maternal supplementation: (1) Fish oil (2.4 g ω-3/day; DHA 1.9 g + EPA 0.22 g), (2) Probiotics, (3) Fish oil + probiotics, (4) Double placebo.	Placebo capsules (medium-chain triglycerides) and/or probiotics (microcrystalline cellulose).	No reduction in risk of physician-diagnosed atopic eczema at 12 or 24 months in any intervention group (all *p* > 0.05).
Figueroa-Garduño et al. [100]	2023	193 Mexican mother–child pairs from the POSGRAD cohort; follow-up of infants to 5 years	Double-blind RCT. Prenatal DHA 400 mg/day from 18 to 22 weeks gestation to delivery. Assessment of maternal urinary arsenic at mid-pregnancy.	Soy/corn-oil placebo group without DHA supplementation.	DHA supplementation decreased early-childhood AD and counteracted the effect of prenatal arsenic exposure.
Niseteo et al. [101]	2024	52 children (1–8 years) with moderate-to-severe AD	4-month—triple-blind RCT. Fish oil syrup (600 mg EPA, 400 mg DHA, 10 mg GLA, 5 µg vit D_3_ daily) + standard AD care	Placebo syrup (MCT oil)	After 4 months, SCORAD significantly decreased in the intervention group vs. no change in placebo. PO-SCORAD, itch, sleep disturbance, and FDLQI improved significantly in the intervention group. No difference in TCS use.

Abbreviations: AA, arachidonic acid; AA/EPA ratio, arachidonic acid to eicosapentaenoic acid ratio; AAD, American Academy of Dermatology; AD, atopic dermatitis; AD-like, atopic dermatitis–like; CBMC, cord blood mononuclear cell; CTLA-4, cytotoxic T-lymphocyte-associated protein 4; DHA, docosahexaenoic acid; DHA-M2, docosahexaenoic acid–induced M2 macrophages; DNCB, 2,4-dinitrochlorobenzene; DNFB, 2,4-dinitrofluorobenzene; EASI, Eczema Area and Severity Index; EPA, eicosapentaenoic acid; EPA-E, eicosapentaenoic acid ethyl ester; FA, fatty acid; FADS2, fatty acid desaturase 2; FDLQI, Family Dermatology Life Quality Index; FK506, tacrolimus; GLA, γ-linolenic acid; HDL, high-density lipoprotein; HR-AD, hairless atopic dermatitis; IgE, immunoglobulin E; IL, interleukin; ISAAC, International Study of Asthma and Allergies in Childhood; LC-PUFA, long-chain polyunsaturated fatty acid; LN, lymph node; LXA_4_, lipoxin A_4_; LTB_4_, leukotriene B_4_; MCT, medium-chain triglyceride; MUFA, monounsaturated fatty acid; PAF, platelet-activating factor; PBMC, peripheral blood mononuclear cell; PGE_2_, prostaglandin E2; PO-SCORAD, Patient Oriented SCORing Atopic Dermatitis; PUFA, polyunsaturated fatty acid; RvE_1_, resolvin E1; SCORAD, SCORing Atopic Dermatitis; SFA, saturated fatty acid; SSS, Symptom Severity Score; TCS, topical corticosteroid; TG, triglyceride; Th, T helper; TGF-β, transforming growth factor-β; TSLP, thymic stromal lymphopoietin; Treg, regulatory T cell; TXB_2_, thromboxane B_2_; ω-3/ω-6, omega-3/omega-6 fatty acid.

### 3.1. Psoriasis

In total, 26 studies evaluating lipid mediators and omega-3 fatty acids were included in the analysis. Within the first evidence stream, eight studies directly examined SPMs in murine models (*n* = 6) or human samples (*n* = 2) (Table 1).

One animal study demonstrated that dietary DHA increased cutaneous levels of several D-series resolvins, protectins, and maresins while simultaneously reducing prostaglandin E_2_ (PGE_2_) and thromboxane B_2_ (TXB_2_) [50]. The remaining preclinical studies investigated exogenous administration of SPMs or their receptor agonists, consistently showing attenuation of imiquimod-induced psoriasiform dermatitis, decreased epidermal hyperplasia, and suppression of pro-inflammatory cytokines such as IL-6, IL-8, and IL-23, accompanied by inhibition of the IL-23/IL-17 axis, along with downregulation of parallel inflammatory pathways involving signal transducer and activator of transcription 1 (STAT1) and NF-κB [45,46,47,48,49]. Human mechanistic and observational investigations further revealed altered profiles of omega-3– and omega-6–derived lipid mediators in psoriatic lesions compared with nonlesional skin and healthy controls, with lesional–control differences exceeding those observed in blood [51,52].

The second evidence stream predominantly concerned the effects of omega-3 fatty acids on psoriasis (Table 2).

Experimental and preclinical studies in cellular and reconstructed skin models showed that DHA, EPA, α-linolenic acid (ALA), and selective free fatty acid receptor 4 (FFA4) agonists improved keratinocyte differentiation, restored lipid-mediator balance, and reduced TH17/TH1-related cytokines [53,55,56,58]. Two studies examining lipoxin A4 receptor agonists in imiquimod-induced murine models reported divergent clinical outcomes, with one study showing no clinical benefit and the other demonstrating significant improvement in psoriasiform skin inflammation [54,57]. Interventional studies in humans demonstrated heterogeneous but overall beneficial outcomes across topical, oral, and intravenous omega-3 PUFA interventions, including reductions in Psoriasis Area and Severity Index (PASI), improvements in quality-of-life measures, and modulation of inflammatory lipid pathways in selected trials. Topical omega-3 PUFA application showed improvement in treated lesions, though differences versus placebo were generally modest [60,64]. Intravenous omega-3 lipid emulsions were associated with rapid clinical response and increased anti-inflammatory lipid mediators [62,65]. Oral omega-3 supplementation yielded variable outcomes, with most trials reporting improvements in clinical outcomes, including PASI reduction, decreased erythema and scaling, and enhanced quality-of-life scores [59,66,67]. Several studies additionally demonstrated modulation of inflammatory lipid mediators and cytokine profiles, supporting anti-inflammatory effects of omega-3 PUFAs [59,68,69]. However, a large randomized controlled trial of high-dose fish-oil ethyl esters showed no clinical benefit over corn oil despite increased serum omega-3 levels [63]. Finally, Mendelian randomization analysis supported a protective association between genetically predicted omega-3 fatty acid levels and psoriasis risk [70].

The included studies on psoriasis exhibited several sources of bias. Preclinical investigations showed high indirectness due to reliance on imiquimod models or in-vitro keratinocyte systems, which do not fully reflect psoriatic pathogenesis. Human mechanistic studies were constrained by small sample sizes, potential confounding, and low detectability of several SPMs, contributing to measurement bias. Clinical interventions were highly heterogeneous, often lacking detailed randomization, blinding, or preregistered protocols, and showed variable dosing and outcome measures, resulting in high risk of performance, detection, and reporting bias.

### 3.2. Acne Vulgaris

In total, seven studies evaluated the role of omega-3 and omega-6 fatty acids as well as PUFA-derived lipid mediators in acne vulgaris; however, none of the included studies directly quantified SPM levels (Table 3).

Evidence from preclinical studies demonstrated that omega-3 supplementation reduced inflammation and comedone formation in acne-induced rats, while fecal microbiota transplantation from omega-3-treated donors confirmed a gut–skin axis mechanism [76]. Observational and genetic evidence indicates that acne patients often present with reduced EPA levels and elevated AA/EPA ratios, while Mendelian randomization analysis supports a protective role of EPA and AA, and a risk-enhancing effect of DGLA, implicating fatty acid desaturase 1 (FADS1) and fatty acid desaturase 2 (FADS2) enzymes in acne pathophysiology [73,77]. Within the interventional evidence stream, five studies evaluating omega-3 fatty acid supplementation showed reductions in acne lesion counts, improvements in patient-reported outcomes, or attenuation of isotretinoin-induced adverse effects [71,72,74,75,76]. Mediterranean diet supplemented with algal DHA/EPA improved acne severity and quality-of-life scores in a 16-week study [75]. In general, omega-3 fatty acids appear to exert protective effects in acne, reducing lesion severity and alleviating treatment-related adverse effects during isotretinoin therapy, although the magnitude of effects varies across studies. Additionally, omega-3 supplementation may beneficially modulate the gut microbiome, highlighting potential systemic mechanisms underlying their therapeutic action.

The included acne studies were limited by small sample sizes, indirect preclinical models, and confounding in human studies. Importantly, none of the studies directly quantified SPMs. Interventional trials were heterogeneous in design, dosing, and outcome measures, resulting in moderate-to-high risk of bias across performance, detection, and reporting domains.

### 3.3. Atopic Dermatitis

As summarized in Table 4, a total of 24 studies addressing AD met the inclusion criteria of this systematic review.

Among these, only two studies directly investigated specialized pro-resolving mediators [79,98]. In preclinical model of AD, intraperitoneal administration of RvE1 attenuated disease severity by suppressing serum IgE, Th1/Th2 cytokine responses, and inflammatory cell infiltration in dose-dependent manner [79]. In interventional human studies, topical application of lipoxin A_4_ in infants with atopic dermatitis resulted in rapid and clinically significant improvements in erythema, pruritus, and inflammatory skin lesions, with efficacy comparable to that of a topical corticosteroid [98].

Regarding omega—fatty acids, a total of 22 eligible studies were identified, including 6 preclinical investigations in animal models, 4 observational studies in humans, and 12 interventional trials. The latter comprised 1 topical intervention [91], 1 intravenous administration [92], 4 oral omega-3 supplementation studies in patients with atopic dermatitis [89,90,94,101], and 6 prenatal supplementation trials assessing the development of allergic diseases, including atopic dermatitis, in infants [93,95,96,97,99,100].

Across preclinical models of atopic dermatitis, dietary or topical administration of omega-3 fatty acids, including DHA and EPA, generally reduced skin inflammation and disease severity, serum IgE levels, inflammatory cell infiltration, and Th2/Th17-related cytokines, while enhancing regulatory immune responses [78,80,81,82,84]. Several studies also demonstrated suppression of keratinocyte-derived thymic stromal lymphopoietin (TSLP) and partial restoration of epidermal barrier function [78,82], whereas selected models showed changes in lipid mediator profiles without clear clinical improvement [83].

Observational and mechanistic human studies indicate that higher maternal or circulating omega-3 long-chain PUFAs, particularly DHA, are generally associated with a lower risk of atopic dermatitis in infants [85,88], whereas elevated omega-6 levels or a higher omega-6/omega-3 ratio increase disease risk [86,87]. In one study, moderate EPA and DHA were linked to lower risk, while omega-3 PUFAs overall and the omega-6/omega-3 ratio showed no consistent association [87]. In patients with atopic dermatitis, FADS2 activity was elevated, resulting in increased omega-6 PUFA production and decreased omega-3 levels [86].

In interventional studies, topical application of EPA/DHA ointment significantly improved clinical outcomes in patients with AD [91], while intravenous infusion of an omega-3–based lipid emulsion reduced disease severity and increased plasma and membrane EPA levels and the EPA/AA ratio [92]. Among studies investigating oral omega-3 supplementation in patients with AD, results were mixed. Some trials reported significant improvements in patient-reported outcomes [89,101], SCORAD scores [94,101], affected skin area, and immunologic markers [94], while other studies found no significant effect on disease severity, topical steroid use, or clinical symptoms [89,90]. In maternal supplementation studies, omega-3 fatty acid intake during pregnancy generally increased DHA and EPA levels in maternal and cord plasma [96,97]. Effects on infant atopic outcomes varied: some trials reported reduced severity of atopic dermatitis [93], lower overall incidence of AD [97,100], reductions limited to IgE-associated eczema [95], or decreased allergen sensitization [93,95], while others found no significant differences in overall incidence or severity of AD during the first 1–2 years of life [93,96,99].

The included AD studies exhibited multiple sources of bias. Preclinical studies were generally indirect, relying on murine or rat models that may not fully reflect human disease. Sample sizes were small, and outcomes were often surrogate or mechanistic, contributing to indirectness and potential measurement bias. Observational human studies were subject to confounding, and variability in fatty acid assessment methods. Interventional trials varied widely in design, dosing, route of administration, and outcome measures. Only two studies directly assessed SPMs, limiting direct mechanistic evidence in humans. Prenatal supplementation trials were generally well-conducted but heterogeneous in timing, dose, and follow-up duration, leading to variable effects on AD incidence or severity.

### 3.4. Factors Contributing to Outcome Differences

Heterogeneity across studies was observed for all three disease entities—psoriasis, acne vulgaris, and atopic dermatitis—arising from multiple sources. Preclinical studies varied in model type (murine, rat, or in vitro keratinocyte/skin substitutes), induction method, and outcome assessment, limiting direct translational comparability. Clinical studies differed in population characteristics, intervention type (oral, intravenous, topical), dose, duration, and timing of supplementation. Outcome measures were diverse, including disease severity scores, lesion counts, cytokine profiles, lipid mediator levels, and quality-of-life indices. Methodological differences, such as randomization, blinding, sample size, and follow-up length, further contributed to variability. Collectively, these factors likely explain the observed heterogeneity in intervention effects and highlight the need for standardized protocols in future trials.

## 4. Discussion

### 4.1. Biosynthesis of Lipid Mediators

Lipid mediators are a collective term referring to signaling molecules derived from the metabolism of PUFAs, which play a key role in regulating the inflammatory response. This broad category includes both pro-inflammatory and pro-resolving molecules, with the overall control of inflammation depending on the balance between two main enzymatic pathways: the cyclooxygenase (COX) and lipoxygenase (LOX) pathways [102]. In the initial step of the inflammatory response, phospholipase A_2_ (PLA_2_) releases PUFAs from membrane phospholipids. These FAs are subsequently metabolized by COX, LOX, and cytochrome P450 enzymes (CYP450) into various classes of lipid mediators. COX metabolizes the omega-6 AA into pro-inflammatory eicosanoids such as prostaglandins and thromboxanes. However, one of the intermediate metabolites can serve as a substrate for 5-lipoxygenase, leading to the production of lipoxins [103].

**Figure 2:**
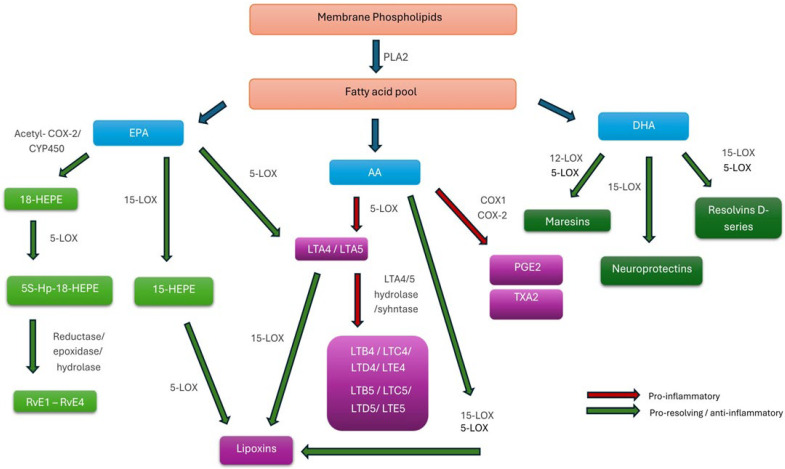
Biosynthetic Pathways of Pro-Inflammatory and Specialized Pro-Resolving Lipid Mediators Derived from Polyunsaturated Fatty Acids. Abbreviations: AA, arachidonic acid; EPA, eicosapentaenoic acid; DHA, docosahexaenoic acid; PLA_2_, phospholipase A_2_; COX-1/COX-2, cyclooxygenase-1/2; acetyl-COX-2, aspirin-acetylated cyclooxygenase-2; CYP450, cytochrome P450 monooxygenase; LOX, lipoxygenase; 5-LOX/12-LOX/15-LOX, 5-, 12-, or 15-lipoxygenase; PG, prostaglandin; PGE_2_, prostaglandin E_2_; TXA_2_, thromboxane A_2_; LT, leukotriene; LTA_4_–LTE_4_, leukotrienes A_4_–E_4_; HEPE, hydroxy-eicosapentaenoic acid; HpEPE, hydroperoxy-eicosapentaenoic acid; RvE_1_–_4_, E-series resolvins; RvD_1_–_6_, D-series resolvins.

The primary precursors of most SPMs are the omega-3 FAs EPA and DHA. Through enzymatic processing by the 5-LOX and 12-LOX pathways, EPA is converted into the E-series resolvins (RvE1–RvE4). In contrast, DHA gives rise to the D-series resolvins (RvD1-RvD6), protectins, and maresins—key lipid mediators that collectively promote the resolution phase of inflammation [104]. Figure 2 illustrates the interconnected biosynthetic pathways that generate both pro-inflammatory and pro-resolving mediators PUFAs.

Dietary omega-3 FAs, primarily EPA and DHA, can be incorporated into membrane phospholipids, thereby increasing the pool of substrates available for the synthesis of SPMs. Consequently, a diet enriched in these FAs enhances the body’s capacity to generate such mediators. The beneficial and protective roles of omega-3 FAs in mitigating chronic inflammatory diseases across various organ systems are well established [105]. Nonetheless, despite decades of research, the therapeutic efficacy of omega-3 supplementation remains a matter of debate, as findings from controlled clinical trials have been inconsistent [106,107,108,109]. The identification of SPMs and their specific receptors has created new opportunities for the development of targeted pharmacological interventions that modulate these pathways. This is of particular interest because pro-resolving mechanisms are anticipated to achieve inflammation control with fewer adverse effects compared to conventional anti-inflammatory agents [104].

### 4.2. Overview of SPMs Classes

SPMs comprise four major families—lipoxins, resolvins, protectins, and maresins [104]. The individual classes of SPMs differ in their functional profiles and timing of action. Each class reaches peak concentrations and displays maximal biological activity at distinct stages of the inflammatory process [104]. Moreover, several SPMs exhibit tissue-specific patterns of distribution, achieving their highest levels in organs characterized by high cellular metabolic activity [110].

**Figure 3:**
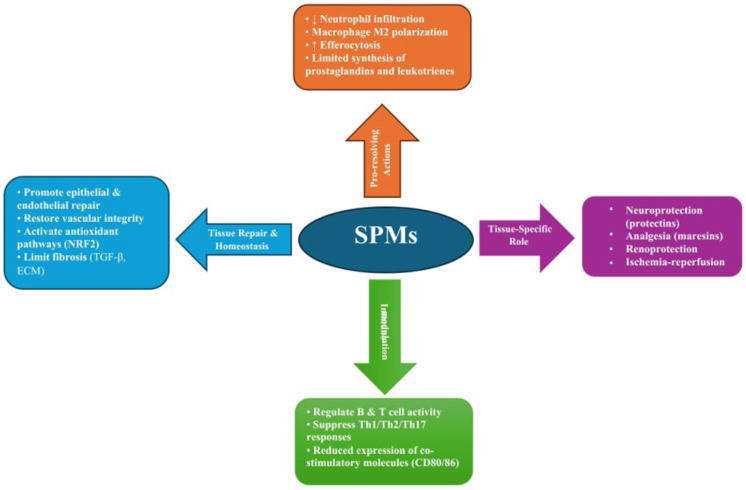
Overview of the pro-resolving and immunomodulatory actions of SPMs, highlighting their roles in resolving inflammation, regulating immune responses, and promoting tissue repair and homeostasis. Abbreviations: SPMs, Specialized Pro-Resolving Mediators; Th1/Th2/Th17, T helper cell subsets type 1, 2, and 17; CD80/CD86, Co-stimulatory molecules expressed on antigen-presenting cells; NRF2, Nuclear Factor Erythroid 2–Related Factor 2; TGF-β, Transforming Growth Factor beta; ECM, Extracellular Matrix; M2, Alternatively activated (anti-inflammatory) macrophage phenotype; ↓, decrease; ↑, increase.

The biological effects of SPMs are mediated through binding to specific receptors, enabling precise modulation of cellular responses [111]. Their actions are complementary and occur in a defined chronological sequence, ensuring a smooth transition from the pro-inflammatory phase to the gradual reduction in inflammatory infiltrates and pro-inflammatory mediators, activation of regenerative processes, tissue repair, and ultimately the restoration of homeostasis [104]. These processes are schematically illustrated in Figure 3, which summarizes the key pro-resolving, immunomodulatory, and other actions of SPMs leading to restoration of homeostasis.

#### 4.2.1. Lipoxins

Lipoxins were the first identified members of the SPMs family [103]. They are produced in multiple tissues via transcellular pathways involving cooperative interactions between migrating polymorphonuclears and epithelial or endothelial cells at the site of inflammation [112]. Lipoxins function predominantly during the early phase of resolution, exerting multiple actions that together promote the termination of inflammation and support tissue homeostasis. Acting primarily through the Lipoxin A4 Receptor/Formyl Peptide Receptor 2 (ALX/FPR2), they limit the production of pro-inflammatory cytokines such as tumor necrosis factor α (TNF-α) and IL-1β, and promote epithelial repair together with the restoration of vascular barrier integrity [113]. Lipoxins modulate both innate and adaptive immune responses to facilitate the resolution of inflammation. They reprogram macrophages toward a proresolving, M2-like phenotype, enhancing efferocytosis and activating transcriptional programs that support tissue repair, while simultaneously reducing neutrophil infiltration [114]. Beyond their effects on innate immunity, lipoxins influence adaptive immune cells by regulating B and T cell responses during the post-resolution phase and limiting the proliferation of memory B cells [12,13]. In addition to their immunomodulatory functions, lipoxins limit fibrosis by modulating transforming growth factor-β (TGF-β) signaling, inhibiting fibroblast proliferation, and reducing extracellular matrix deposition, with evidence that they can reverse established fibrotic lesions in experimental models [115]. In addition, they activate cytoprotective antioxidant pathways, including nuclear factor erythroid 2–related factor 2 (NRF2) signaling, thereby protecting tissues from oxidative stress [116]. Taken together, these actions highlight the central role of lipoxins in orchestrating the transition from inflammation to resolution while preserving tissue integrity and promoting repair.

The metabolism of eicosanoids can be modulated by certain pharmacological agents in ways that favor the formation of lipoxins. For instance, aspirin-induced acetylation or statin-mediated nitrosylation of COX-2 can shift the enzyme’s catalytic function from its typical endoperoxidase activity toward a lipoxygenase-like pathway [117,118]. This functional reprogramming promotes the biosynthesis of epimeric molecules, commonly referred to as aspirin-triggered lipoxins (ATLs). The epimers retain the pro-resolving and anti-inflammatory properties characteristic of native lipoxins but exhibits enhanced metabolic stability and resistance to enzymatic degradation [119]. Other strategies for modulating the lipoxin pathway include the use of stable synthetic analogs, receptor-specific agonists targeting ALX/FPR2, and cell-penetrating peptides that influence intracellular G protein–coupled receptor (GPCR) signaling, all of which enhance lipoxin-mediated resolution and provide potential avenues for therapeutic intervention in chronic inflammatory diseases [120,121].

#### 4.2.2. Resolvins

Resolvins constitute a distinct family of SPMs biosynthesized from omega-3 FAs and are categorized into two main series: the E-series (RvE1–RvE4), derived from EPA, and the D-series (RvD1–RvD6), originating from DHA [120,122,123]. Biologically, resolvins differ from lipoxins in several key aspects. Unlike lipoxins, which act primarily by limiting neutrophil infiltration, resolvins exert broader actions across immune, vascular, and neural systems [120]. They not only dampen inflammation but also actively promote tissue repair, angiogenesis, and neuroprotection, functioning at picomolar to nanomolar concentrations. Aspirin-triggered (AT) epimers, such as AT-RvD1 and AT-RvD3, are generated via acetylation of COX-2 by aspirin, which redirects biosynthetic pathways toward pro-resolving mediators [124,125]. Although resolvins generally signal through distinct receptors, not all have fully characterized binding partners. Some can engage receptors shared with lipoxins, interacting with alternative binding sites and functioning as biased agonists, thereby eliciting divergent intracellular signaling pathways [126]. Collectively, these account for their therapeutic potential in chronic inflammatory diseases, infections, asthma, atherosclerosis, and neurodegenerative disorders.

#### 4.2.3. Protectins

Protectin D1 (PD1), also known as neuroprotectin D1 (NPD1) in neural tissues, is a DHA–derived SPM whose actions depend on tissue context and cell type [127]. In the nervous system, NPD1 promotes neuronal survival by counteracting oxidative stress and enhancing apoptosis [127]. Outside the nervous system, PD1 functions as an immunoresolvent, limiting cytokine release, reducing neutrophil infiltration, and promoting macrophage-mediated clearance [127]. It also exhibits antiviral activity and contributes to tissue repair [128]. The dual capacity of PD1 to suppress inflammation while enhancing cell survival highlights its relevance as a potential therapeutic mediator in neurodegenerative, retinal, and ischemic pathologies.

#### 4.2.4. Maresins

Maresins are SPMs derived from DHA, possessing both anti-inflammatory and pro-resolving properties. The group comprises three primary forms: maresin 1 (MaR1), maresin 2 (MaR2), and maresin conjugates in tissue regeneration (MCTR) [129]. These mediators are produced by 12-LOX in macrophages and dendritic cells, where they promote efferocytosis and facilitate the resolution of inflammation. Maresins also suppress the expression of pro-inflammatory cytokines and limit polymorphonuclear leukocyte infiltration [130]. Notably, unlike other classes of pro-resolving mediators, maresins exhibit analgesic effects [131], highlighting their unique role in both inflammation resolution and pain modulation.

### 4.3. Lipids as a Structural and Bioactive Regulators of Cutaneous Homeostasis

The skin is a lipid-rich organ, and its barrier function critically depends on the composition of PUFAs [132]. Both omega-3 and omega-6 PUFAs are integral components of epidermal phospholipids and ceramides. Omega-6 PUFAs, particularly linoleic acid, facilitate ceramide synthesis, thereby enhancing barrier function and reducing transepidermal water loss (TEWL) [133], whereas omega-3 PUFAs exhibit anti-inflammatory and antioxidant properties, modulating cutaneous immune responses and protecting against environmental stressors such as UV radiation and pollution [134,135]. Beyond structural roles, these FAs serve as precursors for a diverse array of bioactive lipid mediators, including SPMs [2].

Dysregulation of PUFAs metabolism and chronic inflammation are central features in the pathogenesis of persistent dermatological conditions, including AD, psoriasis, and acne [56,136,137]. Altered ratios of omega-6 to omega-3 PUFAs can exacerbate pro-inflammatory signaling, leading to sustained epidermal barrier dysfunction, increased TEWL, and heightened susceptibility to environmental insults [138,139]. Moreover, chronic inflammatory states are often accompanied by disturbances in lipid homeostasis, including impaired ceramide synthesis and altered phospholipid composition, which further compromise barrier integrity and skin hydration [140]. These observations have stimulated increasing interest in dietary interventions as modulators of disease course, with omega-3 PUFAs supplementation demonstrating benefits in reducing inflammatory markers, restoring lipid balance, and improving clinical outcomes in chronic inflammatory skin disorders [58,67]. Enhanced availability of omega-3 PUFAs facilitates the enzymatic conversion of EPA and DHA into their respective SPMs families, thereby augmenting the intrinsic capacity of the host to generate pro-resolving lipid mediators [141,142]. Accumulating evidence shows that omega-3 PUFAs can also directly modulate immune regulation by promoting expansion and functional activity of Tregs, shifting immune balance toward resolution rather than inflammation. For instance, in a murine model of atopic dermatitis, administration of DHA enhanced the generation of CD4^+^Foxp3^+^ Tregs and increased anti-inflammatory IL-10/TGF-β signaling, reducing skin inflammation [80]. Another example comes from a 3D psoriatic skin model, where EPA treatment decreased IL-17A–secreting T cells and increased FOXP3^+^ Treg populations, resulting in normalized keratinocyte proliferation and reduced inflammatory mediator levels [58]. Consequently, an increased dietary or systemic supply of omega-3 FAs serves not only as the substrate pool but also promotes the biochemical milieu favorable for the resolution of inflammation. Targeting PUFAs composition through nutrition represents a promising adjunct strategy for managing chronic skin diseases by addressing both inflammation and lipid metabolic imbalance.

### 4.4. Psoriasis

#### 4.4.1. Lipid Dysregulation in Psoriasis

Psoriasis is widely recognized as a disease predominantly driven by Th1/Th17 immune responses, with elevated levels of cytokines such as IL-17A, IL-17F, IL-22, IL-23, TNF-α, and IFN-γ playing central roles in its pathogenesis [143]. While traditionally considered an immune-mediated disorder developing in genetically predisposed host, increasing evidence highlights a crucial contribution of lipid metabolism to its pathogenesis. Psoriatic skin exhibits altered lipid composition, including disturbed levels of ceramides, free FAs, and eicosanoids, which can impair barrier integrity and amplify inflammatory cascades through the AA and prostaglandin pathways [51,140]. Genes involved in lipid metabolism, such as *PLA2G4E*, *ELOVL*, and *FABP* family members, have been shown to be dysregulated in psoriatic skin, indicating that altered lipid processing may play a role in disease pathogenesis [140,144,145]. These genetic variations may alter the synthesis or turnover of structural and signaling lipids, thereby facilitating immune activation and perpetuating chronic inflammation. Recent research has provided evidence that higher genetically predicted omega-3 FAs levels are associated with a reduced risk of psoriasis, thereby strengthening the evidence that omega-3 FAs may play a direct, biologically meaningful role in modulating psoriasis susceptibility [70]. Thus, the interplay between genetic predisposition and lipid dysregulation represents a key but still incompletely understood component of psoriasis pathogenesis.

These genetic and metabolic disturbances are reflected in a distinct lipidomic profile observed in individuals with psoriasis, characterized by an imbalance between unsaturated FAs classes [146]. The observed shift toward a higher proportion of monounsaturated species suggests altered desaturase activity and disrupted metabolic regulation, while the concurrent reduction in polyunsaturated fractions and a distorted omega-6 to omega-3 equilibrium may contribute to the pro-inflammatory milieu typical of psoriasis. Current evidence indicates that patients with psoriasis display decreased levels of omega-3 PUFAs and elevated omega-6 FAs in both serum and erythrocyte membranes, correlating with higher inflammatory markers such as C-reactive protein (CRP) and IL-6, as well as greater disease severity [146,147]. Studies also suggest that an increased omega-6/omega-3 ratio and elevated saturated FAs content may promote systemic inflammation, metabolic disturbances, and comorbidities including obesity, hepatic dysfunction, and cardiovascular risk [147,148].

#### 4.4.2. Evidence for Omega-3 Fatty Acids Supplementation

Investigations into the therapeutic potential of omega-3 FAs supplementation, including topical applications, in psoriasis began in the late 1980s [59,60]. Early studies demonstrated encouraging outcomes in small patient cohorts; however, these findings were subsequently scrutinized, partly due to the lack of double-blind study designs. In the following decades, further evidence emerged from randomized, double-blind trials; however, the findings remained heterogeneous, primarily due to variations in dosage, study duration, and selected clinical endpoints. Two small studies evaluating the efficacy of a topical formulation containing omega-3 FAs were conducted in comparison with a placebo cream, applied simultaneously to matched lesions within the same patients [60,64]. The first report, involving a cohort of 11 patients with psoriasis, demonstrated objective improvement and suggested the superiority of the omega-3 cream over the placebo [60]. However, a subsequent larger, multicenter study found that general improvement occurred with both formulations, but neither formulation was significantly more effective than the other [64]. The vast majority of available data come from clinical trials investigating the impact of oral supplementation with omega-3 FAs on the course of psoriasis. Most studies demonstrated a generally positive effect on disease activity and associated symptoms, including pruritus; however, differences were not always statistically significant when assessed using objective measures such as the PASI score [67,68]. Interpretation of these results is challenging due to the heterogeneity of study designs, with dosages ranging from 2.6 to 3.6 g/day of EPA ± DHA and treatment durations from 12 weeks to 12 months [61,67]. According to the findings reported by Petrovic et al. [69], the beneficial effect of omega-3 PUFAs supplementation in plaque psoriasis appears to be mediated by anti-inflammatory mechanisms, as evidenced by alterations in immune cell populations and cytokine profiles that correlated with clinical improvement. Notably, two studies assessed the effects of intravenous monotherapy with omega-3–enriched lipid emulsions in comparison to omega-6–based formulations [62,65]. Both studies reported a significant reduction in PASI scores alongside an increase in omega-3–derived eicosanoids, suggesting a distinct therapeutic effect attributable to high daily doses of EPA and DHA. Furthermore, omega-3–enriched parenteral nutrition is recognized as a key component of postoperative care and the management of critically ill patients, as emphasized in current guidelines on intravenous lipid emulsions [149]. Although the meta-analysis assessing fish oil supplementation in psoriasis indicated no statistically significant improvement in PASI scores compared to controls (MD −0.28; 95% CI −1.74 to 1.19; I^2^ = 57%), individual studies within the analysis reported trends toward improvement in body surface area, as well as reductions in erythema, scaling, and induration, particularly when fish oil was combined with adjunctive therapies [150]. Morin et al. demonstrated in in vitro models of psoriatic and healthy skin that supplementation with DHA or EPA—exerts notable anti-inflammatory and homeostatic effects. DHA reduced abnormal keratinocyte differentiation, lowered pro-inflammatory mediators (PGE_2_, 12-HETE, TNF-α), and rebalanced PPAR expression, thereby attenuating psoriatic characteristics [55]. EPA supplementation enriched epidermal omega-3 lipid content, increased anti-inflammatory metabolites (PGE_3_, 12-HEPE, EPEA), and decreased omega-6–derived products, restoring lipid and inflammatory equilibrium [58]. Moreover, DHA supplementation may also regulate epidermal keratinization processes [53]. In approximately 75% of psoriasis cases, a deletion of two late cornified envelope genes (*LCE3B–C*) is observed [151]. DHA and other omega-3 FAs have been shown to act as potent modulators of epidermal inflammation and differentiation in psoriasis. An open question remains regarding the precise pathways through which omega-3 FAs exert their beneficial effects in alleviating psoriasis. It has been postulated that these mechanisms involve competition as substrates for COX, increased production of SPMs, and modulation of the FFA4 receptor [54,57]. Two studies investigating a synthetic FFA4 agonist yielded conflicting results. A study led by Wannick et al. [54] reported no improvement in a murine model of psoriasis. In contrast, Son et al. observed both clinical improvements and reductions in pro-inflammatory mediators, confirming that the effects were mediated via the FFA4 receptor [57]. Notably, the two studies differed in the route of administration (oral versus parenteral, respectively), which may account for the observed discrepancies.

#### 4.4.3. Evidence for SPMs in Psoriasis

Although the clinical effects of omega-3 supplementation may result from multiple mechanisms, experimental evidence suggests that enhanced SPMs biosynthesis and modulation of the IL-23/IL-17 axis play a key role. The first direct evidence supporting this theory dates back to 2011, when a research team demonstrated that lipoxin A4, in vitro, inhibits the expression of IL-6 and TNF-α in both keratinocytes and fibroblasts [45]. In line with these findings, treatment with a synthetic LXA_4_ agonist markedly reduced psoriatic inflammation in a murine model [46]. Similarly, Xu et al. demonstrated that pretreatment with RvD1 attenuated imiquimod (IMQ)-induced psoriasiform dermatitis by activating ALX/FPR2, leading to the suppression of the IL-23/IL-17 inflammatory axis [47]. A key downstream pathway implicated in post-receptor signaling involves high-mobility group box protein 1 (HMGB1), a pro-inflammatory mediator that amplifies the IL-23/IL-17A pathway, promotes keratinocyte activation, and facilitates the recruitment of immune cells to psoriatic lesions [152]. Elevated HMGB1 levels have been correlated with disease severity and the persistence of inflammatory responses in psoriasis [152].

Several other SPMs, including RvE1 and PD1, have also been identified as potent modulators of psoriatic inflammation [48,49]. Their administration reduces epidermal scaling, plaque thickness, and erythema, reflecting restoration of epidermal homeostasis. In addition, these mediators downregulate key cytokines involved in psoriasis pathogenesis. In preclinical models, SPMs have been shown to downregulate IL-17 and TNF-α expression, suppress the Th17 axis, reduce immune cell infiltration, and mitigate psoriasiform skin inflammation, highlighting their potential as pro-resolving therapeutic agents [47,48]. In murine models, omega-3 FA supplementation increased cutaneous levels of pro-resolving mediators, including RvD5, protectin DX, and MaR2, while EPA reduced pro-inflammatory PGE_2_ and TXB_2_ [50]. These findings indicate that omega-3 supplementation can simultaneously enhance resolution pathways and suppress pro-inflammatory eicosanoids. RvD1 and RvD5 reduce keratinocyte-driven inflammation by downregulating IL-24 and S100A12, suggesting their potential to attenuate psoriatic plaque formation and limit immune cell recruitment in affected skin [52].

Human data on SPMs in psoriasis remain limited and derive from only two studies [51,52]. These investigations compared lipid mediator profiles in the serum and skin biopsies of patients with psoriasis versus healthy controls, and additionally assessed differences between lesional and non-lesional skin within affected individuals. Within psoriatic lesions, an increase in DHA–derived metabolites (14-HDHA and 17-HDHA) was observed, suggesting partial activation of DHA-associated resolution pathways [52]. Conversely, metabolites originating from EPA, such as 14,15-epoxyeicosatetraenoic acid (14,15-EpETE) and 17,18-epoxyeicosatetraenoic acid (17,18-EpETE), were scarcely detectable or absent [51]. This pattern suggests a disruption or insufficiency in the EPA-derived lipid mediator pathway, leading to an incomplete activation of resolution-phase processes. Several omega-3 and omega-6 metabolites found in the skin were also detected in the bloodstream, although their differences were less marked. Higher serum hydroxyeicosatetraenoic acid (HETE) levels, an oxylipin derived from AA, in healthy individuals and comparable RvD2 detection across groups suggest that lipid mediator dysregulation in psoriasis is largely confined to the skin, making serum not representative for local inflammatory activity [51]. Clinically, localized disruption of lipid mediator homeostasis may sustain chronic skin inflammation despite modest systemic involvement. Thus, circulating biomarkers may have limited diagnostic value, while interventions that restore lipid balance—such as omega-3 supplementation, LOX/COX modulation, or correction of lipid oxidation and bile acid metabolism—could support resolution of psoriatic inflammation.

In summary, current evidence underscores the pivotal yet underexplored role of specialized SPMs and omega-3–derived lipids in psoriasis. Alterations in cutaneous lipid mediator networks—characterized by a relative deficiency of omega-3–derived pro-resolving metabolites and dominance of omega-6–driven inflammation—appear to impair resolution pathways and perpetuate chronic skin inflammation. These findings highlight the therapeutic potential of targeting lipid metabolism and enhancing SPMs biosynthesis as complementary strategies for restoring immune and epidermal homeostasis in psoriasis.

### 4.5. Hidradenitis Suppurativa

HS is a chronic, relapsing inflammatory skin disease characterized by a pathogenic triad of aberrant immune activation, dysregulated metabolic signaling, and impaired epithelial differentiation [153]. It predominantly affects intertriginous areas, including the axillae, groin, inframammary, and perianal regions, where it manifests as recurrent painful nodules, abscesses, and draining sinus tracts [154]. The disease follows a debilitating course and profoundly impacts multiple aspects of patients’ lives, including occupational functioning, social interactions, and sexual health, often resulting in significant psychosocial distress and social isolation [155]. HS is frequently associated with a range of systemic comorbidities, most notably components of metabolic syndrome. Estimates suggest that up to half of all patients with HS meet the diagnostic criteria for metabolic syndrome, including obesity, hypertension, dyslipidemia, and insulin resistance [156]. The chronic inflammatory state contributes to a markedly increased overall health burden. Consequently, individuals with HS experience a substantially reduced life expectancy, with some studies reporting a decrease of approximately 15 years compared to the general population [157].

This elevated morbidity and mortality underscore the systemic nature of the disease. HS is characterized by a dominant Th1/Th17 immune response, with lesional skin exhibiting elevated levels of pro-inflammatory cytokines including TNF-α, IL-1β, IL-6, IL-17, and IL-23 [154]. Existing evidence indicates that HS is characterized by both systemic lipid dysregulation and a localized imbalance of cutaneous lipid mediators. Lesional skin exhibits impaired regulation of sphingolipid-metabolizing enzymes, with a shift toward catabolic pathways [157]. Recent lipidomic analyses in HS have revealed systemic alterations in lipid metabolism, particularly dysregulation of sphingolipid pathways, and elevated levels of pro-inflammatory oxylipins—such as HETEs and DHETs—correlating with disease activity [158]. Although systemic changes were less pronounced than those observed in lesional skin [159], the study’s methodological design ensured that the detected changes reflected a disease-specific lipid imbalance independent of metabolic comorbidities.

To date, direct evidence concerning SPMs in HS is lacking; therefore, this section aims to summarize the available data on pro-inflammatory lipid mediators and identify existing gaps in the current literature. Lipidomic profiling of lesional HS skin revealed a distinct pro-inflammatory shift, characterized by elevated levels of 5-LOX–derived metabolites from AA—notably 5-HETE, 5-oxo-eicosatetraenoic acid (5-oxo-ETE), and leukotriene B_4_ (LTB_4_) —accompanied by reduced concentrations of anti-inflammatory products generated via 12-LOX and 15-LOX pathways from EPA and DHA [133]. Although the study did not directly measure SPMs, the observed reduction in 15-hydroxyeicosapentaenoic acid (15-HEPE)—a key precursor of E-series resolvins—indirectly suggests a diminished capacity for SPMs biosynthesis. This deficiency may contribute to the chronic, non-resolving Th1/Th17-driven inflammatory phenotype characteristic of HS, while highlighting the potential underrepresentation of pro-resolving pathways in the lesional lipidome. Taken together, these findings underscore a clear pro-inflammatory lipid signature in HS but also point to several important gaps in knowledge that require further investigation. First, the detailed composition of FAs in both lesional and circulating compartments of HS patients—particularly the balance between omega-3 and omega-6 species—remains poorly characterized. Second, the specific contributions of pro-resolving lipid mediators to HS pathophysiology have been largely unexplored, with the majority of existing data focusing on pro-inflammatory eicosanoids. Third, it remains unclear whether lipid dysregulation acts as a primary driver of HS pathogenesis or arises secondarily as a consequence of chronic inflammation. Finally, therapeutic strategies targeting lipid pathways—such as dietary modulation of FAs or pharmacologic manipulation of lipid mediators—are urgently needed, given that outcomes achieved with currently available treatments, which primarily focus on the inhibition of pro-inflammatory cytokines, remain largely unsatisfactory.

### 4.6. Acne Vulgaris

Acne vulgaris is a common chronic dermatological condition involving the pilosebaceous unit. It is one of the most prevalent diseases worldwide, affecting approximately 9.4% of the global population [160], and its incidence appears to be increasing [74]. The condition may manifest with non-inflammatory lesions (open and closed comedones) as well as inflammatory lesions (papules, pustules, and nodules) [161]. Although acne typically begins during adolescence and predominantly affects young males, it may also develop in adults, among whom it is more frequently observed in women. The pathogenesis of acne involves increased follicular keratinization, excess production of sebum, inflammation, and colonization of the hair follicles by Cutibacterium acnes [161]. Genetic and ethnic factors influence acne susceptibility; however, increasing incidence has been associated with the adoption of Western lifestyles, which are characterized by low intake of PUFAs and high consumption of saturated fats and refined carbohydrates [162]. Acne affects daily functioning, causing feelings of embarrassment, anxiety, and discomfort [163].

Initially, inflammation was described as occurring in the later stages of acne, accompanying clinical lesions such as papules and pustules. More recent studies, however, indicate that inflammation is also present in the early stages of acne development [77,164]. Moreover, evidence suggests that inflammation may precede hyperkeratinization of the pilosebaceous unit. This is supported by the observation of elevated levels of CD3^+^ and CD4^+^ T cells in the perifollicular and papillary dermis, as well as an increased number of macrophages in clinically unaffected skin in acne patients [165,166].

Scientific studies indicate possible lipid abnormalities in patients with acne. Sobhan et al. demonstrated significantly higher total cholesterol levels, particularly among male patients with acne [167]. Other lipid parameters (triglicerydes, LDL, and HDL) were higher in the acne group than in controls, but the differences were not statistically significant. Jiang et al. reported significantly elevated levels of total cholesterol, LDL, and lipoprotein A in acne patients compared to control groups, with male patients also showing higher triglyceride levels [168]. Research suggests that certain lipid components and their ratios—particularly the omega-6/omega-3 and PUFA/MUFA ratios—may have a causal influence on acne risk [137]. Gokdemir et al. found statistically higher serum triglyceride concentrations in patients with acne [169]. Although the existing data present inconsistent findings, they collectively highlight potential directions for future large-scale investigations.

The enzyme lipoprotein lipase (LPL) catalyzes the hydrolysis of triglycerides in plasma triglyceride-rich lipoproteins at the surface of capillary endothelial cells. Its expression has also been detected in skin appendages, implying that sebaceous glands possess the ability to sequester FAs, which may be associated with the pathogenesis of acne [73]. The angiopoietin-like proteins (ANGPTLs) 3 and 4 are the main regulators of plasma lipid metabolism due to their inhibitory effect on LPL. Secretory PLA2, hydrolyzes phospholipids, generating free FAs and lysophospholipids, which exacerbate the inflammation [73]. Aslan et al. demonstrated increased activity of sPLA_2_ and elevated AA/EPA and dihomylo-γ-gamma-linoleic acid/eicosapentaenoic acid (DGLA/EPA) ratios in patients with acne. Significantly higher activity of LPL was also observed in the same cohort [73]. The activities of both enzymes in sebaceous glands generate free FAs that can fuel inflammation leading to acne pathogenesis.

Recent studies have begun to elucidate the relationship between lipids and inflammation in acne, with particular emphasis on PUFAs [73,74,75,77,160,168,169]. Among these studies, omega-3 supplementation was associated with an improvement in acne lesions [71,137,165,167], and with a beneficial effect on gut microbial diversity [76]. However, current evidence regarding PUFA-enriched diets remains limited due to small sample sizes, heterogeneous interventions, and inconsistent outcome measures. Moreover, during systemic acne therapy with isotretinoin, omega-3 FAs were shown to reduce some of the drug’s adverse effects [74]. In contrast to omega-3, the role of omega-6 FAs in acne remains unclear. γ -linoleic acid (GLA) appears to improve acne lesions [72], whereas Zhang et al. demonstrated that DGLA may exacerbate acne [77]. Additionally, AA and EPA were identified as protective factors against the development of inflammation in acne [77]. In acne vulgaris patients higher levels of AA/EPA and DGLA/EPA ratios were observed [73]. These findings suggest that a higher dietary intake of omega-3 FAs, combined with reduced consumption of saturated fats, may exert a beneficial therapeutic effect in the management of acne. Furthermore, Zhang et al. identified several SNPs within the *fatty acid desaturase 1 (FADS1)* and *fatty acid desaturase 2 (FADS2)* genes. These variants may affect the enzymatic activity of FADS1 and FADS2 in PUFA metabolism, thereby contributing to the worsening of acne symptoms [77]. Personalized approaches based on lipid and genomic profiling could therefore help optimize therapeutic outcomes in acne patients.

Acne vulgaris is associated with a mixed but predominantly Th1/Th17-type immune response, characterized by elevated levels of pro-inflammatory cytokines such as IL-1β, IL-6, IL-8, IL-17 and TNF-α, which contribute to follicular inflammation, neutrophil infiltration and lesion formation [170,171]. Emerging evidence suggests that SPMs can limit pro-inflammatory cytokine production and leukocyte infiltration, thereby promoting resolution of inflammation and offering potential therapeutic benefits in acne. So far, no direct link has been established between SPMs and acne development. However, the involvement of inflammatory processes in the early stages of acne formation and comedogenesis, together with the beneficial effects of omega-3 FAs on the course of the disease, suggests that inadequate resolution of inflammation and disturbances in lipid mediator homeostasis may contribute to the pathogenesis of acne vulgaris. While SPMs represent a promising new direction for acne research, the beneficial effects of omega-3 FAs cannot be explained solely by their conversion to SPMs. Omega-3 FAs exert multifaceted biological actions among which the bactericidal activity of long chain PUFAs against Cutibacterium acnes and Staphylococcus aureus appears particularly relevant to acne pathogenesis [39]. Moreover, omega-3 FAs provide alternative substrates for LPL and sPLA_2_, potentially reducing the synthesis of pro-inflammatory mediators. Thus, understanding how SPMs and PUFA-derived lipid mediators contribute to the regulation of inflammation and microbial balance in acne may open new therapeutic perspectives.

### 4.7. Atopic Dermatitis

Similar to psoriasis, AD is characterized by an imbalance between pro- and anti-inflammatory lipids, prompting investigation into the role of omega-3 FAs and SPMs in this condition. AD constitutes a major global dermatological health burden, affecting approximately 10–20% of children and up to 3% of adults [172]. Multifactorial pathogenesis of AD encompasses genetic predisposition, epidermal barrier dysfunction, immune dysregulation, and environmental determinants [18,101]. Of particular note, the prevalence of AD has shown a progressive increase over recent decades, a trend partly attributed to changes in dietary habits, particularly the shift toward higher consumption of omega-6 PUFAs and the resulting imbalance in the omega-6 to omega-3 ratio [173]. Growing evidence indicates that disruption of the omega-6 to omega-3 ratio contributes to dysregulated eicosanoid signaling and persistent inflammation [174].Immunologically, acute AD is primarily mediated by a Th2-dominant response, with heightened expression of IL-4, IL-5, IL-13, IL-31, and occasionally IL-22, while chronic or treatment-resistant lesions frequently involve additional activation of Th1, Th17, and Th22 pathways [175].

At the molecular level, omega-3–derived lipid mediators exert pleiotropic immunomodulatory and barrier-stabilizing effects on the cutaneous microenvironment characteristic of AD [78,80,81,82]. EPA and DHA compete with AA for COX and LOX pathways, thereby reducing the generation of pro-inflammatory eicosanoids such as PGE_2_ and LTB_4_ [81,83]. In parallel, the conversion of EPA and DHA into specialized pro-resolving lipid mediators actively terminates inflammation through the downregulation of Th2- and Th17-driven cytokines (IL-4, IL-5, IL-13, IL-17A) and the suppression of IgE synthesis [80,82]. These mediators also enhance regulatory T-cell (Treg) activity, promote macrophage M2 polarization, and restore epidermal barrier integrity by normalizing ceramide synthesis and reducing TEWL [44,80]. Given the dominant Th2 cytokine milieu in acute AD, which expands to include Th17 and Th22 responses in chronic stages, SPM-mediated downregulation of IL-4, IL-13, and other inflammatory mediators offers a promising mechanism to rebalance immune responses, support barrier repair, and potentially reduce pruritus and allergen sensitivity. Indeed, impaired generation of SPMs has been proposed as a contributing factor to the chronic, non-resolving inflammation in AD, and restoration of pro-resolving lipid mediator pathways is increasingly viewed as a potential novel therapeutic strategy [176].

Dietary modulation of PUFA intake and direct SPMs administration have been explored as therapeutic approaches for AD [78,79,81,82,83,89,90,92,93,96,97,99,101]. The following studies, summarized in Table 4, encompass both clinical and preclinical investigations exploring the influence of omega-3/omega-6 FAs and SPMs on the onset and severity of AD. These studies evaluate diverse interventions—including maternal supplementation during pregnancy, direct oral intake of EPA/DHA, topical formulations, and experimental administration of SPMs analogues—in both human cohorts and animal models. Experimental studies consistently demonstrate that EPA- and DHA-derived mediators attenuate Th2/Th17 inflammation, reduce IgE and histamine levels, and restore epidermal lipid balance, thereby improving barrier function [78,79,80,81,82,84]. These effects are further supported by data showing enhanced Treg activity and suppression of pro-inflammatory cytokines following SPMs or omega-3 administration [80,96].

In contrast, clinical findings are more heterogeneous: while several randomized controlled trials and maternal supplementation studies report reduced IgE-associated eczema [95], decreased allergen sensitization, or milder disease severity [93], others find no significant preventive or therapeutic effects [89,90,96,99]. Recent trials further substantiate both the potential and the limitations of omega-3 interventions. Supplementation with DHA/EPA in adults and children with AD has been shown to significantly reduce SCORAD scores and improve patient-reported outcomes [94,101], and maternal omega-3 intake during pregnancy was associated with a lower incidence of IgE-mediated eczema in infants [95]. In the context of SPMs and omega-3/omega-6 FAs in AD, there is emerging evidence that maternal DHA intake may modify the increased risk of AD associated with prenatal arsenic exposure [100]. However, other well-controlled studies demonstrated no reduction in eczema risk despite clear biochemical evidence of increased maternal and infant DHA/EPA levels and dampened cytokine responses [96,99]. Such discrepancies may be attributed to heterogeneity in population risk profiles, baseline dietary patterns, genetic factors, and timing or dosage of supplementation. Recent meta-analytic evidence indicates that although maternal omega-3 PUFAs supplementation during pregnancy does not significantly reduce the overall incidence of eczema in offspring, it may confer a potential subgroup benefit for IgE-associated eczema in children under three years of age, highlighting age-dependent and immunophenotype-specific effects of prenatal omega-3 exposure [177]. Overall, while omega-3 FAs exhibit immunomodulatory and barrier-stabilizing properties, the inconsistency of clinical outcomes precludes universal recommendations and highlights the multifactorial nature of AD pathophysiology.

Consequently, attention has increasingly shifted toward SPMs, which exert potent anti-inflammatory and pro-resolving actions at nanomolar concentrations [124]. Their ability to actively terminate inflammation and promote tissue homeostasis has drawn researchers’ attention to the development of stable analogues or receptor agonists for clinical application. To date, the only human study in AD utilizing an SPMs-based approach evaluated a topical 15(R/S)-methyl-lipoxin A_4_ cream in infants with acute facial eczema, which significantly improved erythema, pruritus, and lesion severity within days [98]. Moreover, the cream demonstrated comparable efficacy to medium-potency corticosteroids without adverse effects; however, the relapse rate at 40 days was higher than in the control group treated with mometasone [98]. These findings suggest that targeting the SPM pathway may represent a promising, mechanism-driven therapeutic strategy distinct from conventional anti-inflammatory treatments.

### 4.8. Integrated Summary of Findings and Overall Limitations

Across psoriasis, AD, acne, and HS, the findings of this systematic review demonstrate that disturbances in lipid mediator networks—particularly imbalances between omega-3– and omega-6–derived metabolites—are consistently associated with aggravated inflammation, impaired barrier function, and dysregulated immune responses. Hidradenitis suppurativa, psoriasis, and acne vulgaris are predominantly Th1/Th17-driven, with elevated IL-17A, IL-17F, IL-23, TNF-α, and IFN-γ promoting keratinocyte activation, neutrophil recruitment, and chronic inflammation, whereas atopic dermatitis is primarily Th2-mediated, with IL-4, IL-5, IL-13, and IL-31 driving IgE production, barrier dysfunction, and allergic sensitization. SPMs modulate immune responses in a context-dependent manner, targeting the dominant T-helper cell profile: suppressing IFN-γ and TNF-α in Th1-driven diseases, downregulating IL-4, IL-5, and IL-13 in Th2-mediated disorders, and attenuating IL-17/IL-23 signaling and neutrophil-driven inflammation in Th17-skewed conditions. Across all contexts, SPMs enhance Treg activity and promote macrophage M2 polarization, providing a conserved mechanism for inflammation resolution and tissue repair. Human data remain limited but broadly align with experimental findings, suggesting that insufficient SPMs biosynthesis or precursor availability may contribute to chronic, non-resolving inflammation. Interventional studies involving omega-3 supplementation show variable but generally favorable outcomes in psoriasis, acne, and AD, particularly regarding patient-reported symptoms. Evidence in HS is minimal and indirect. Taken together, the evidence suggests that lipid mediators derived from PUFAs act as central regulators of immune resolution in the skin.

This body of evidence has notable limitations, including the scarcity of human studies, small cohorts, and heterogeneous interventions. Many trials depend on non-standardized or subjective outcomes, and dietary studies may be influenced by confounding factors, including lifestyle and genetic susceptibility. Moreover, mechanistic in vitro and animal data may not adequately reflect human physiology, reducing translational relevance. The reliance on mechanistic and animal studies, together with the probable underrepresentation of studies reporting null or negative outcomes, limits confidence in the estimated therapeutic benefits of omega-3 fatty acids and SPMs in humans.

This review also has methodological limitations. Only one database (MEDLINE/PubMed) was searched, which may have led to missed studies published elsewhere. A major limitation of this review is the lack of a formal risk-of-bias assessment. This was due to the substantial heterogeneity and mechanistic nature of the included evidence, which comprised in vitro studies, animal models, and exploratory human research—study types for which no unified risk-assessment framework exists. Additionally, highly variable methodologies, small sample sizes, and inconsistent outcome reporting across studies prevented meta-analysis, assessment of reporting bias, and formal evaluation of certainty of evidence. Moreover, considerable variability in analytical techniques for quantifying lipid mediators—including differences in sample preparation, detection platforms, and quantification methods—further limits comparability across studies and introduces potential measurement inconsistency. Additionally, publication bias could not be evaluated, and certainty of evidence was not assessed due to the nature of the included data.

Clinically, omega-3 supplementation may offer complementary benefits in inflammatory skin diseases, although current evidence is insufficient for formal recommendations. SPM-based therapies, particularly stable analogues and receptor agonists, represent a promising yet still emerging therapeutic direction. Future research should focus on well-designed clinical trials with standardized clinical and biochemical endpoints, comprehensive lipidomic profiling, and mechanistic work on SPM analogues and agonists, as these agents offer the most realistic route toward therapeutic translation.

## 5. Conclusions

Dysregulated lipid metabolism—particularly imbalances in omega-3 and omega-6–derived mediators—plays a central role in the pathogenesis of psoriasis, HS, acne vulgaris and AD. SPMs act at the interface of lipid metabolism, immune regulation, and tissue repair, facilitating the active resolution of cutaneous inflammation. Accordingly, omega-3 FAs and their downstream SPMs represent promising adjunctive therapies capable of restoring inflammatory balance and promoting tissue homeostasis. While mechanistic and early clinical data on SPMs are compelling, further research is needed to comprehensively characterize SPMs profiles in inflammatory skin disorders. Additional studies are also required to evaluate the therapeutic potential of SPM analogues and receptor agonists, particularly given their superior stability and receptor specificity compared with endogenous mediators. The limited available human data suggest that deficiencies in omega-3–derived pro-resolving mediators, together with a shift toward omega-6–dominant inflammatory profiles, may contribute to disease chronicity and severity. This imbalance highlights the relevance of targeting lipid mediator pathways as a complementary strategy to conventional anti-inflammatory therapies. To sum up, omega-3 FAs and SPMs represent promising adjuncts for managing several inflammatory skin conditions through their capacity to rebalance inflammatory pathways and promote resolution pathways.

## Data Availability

All data analyzed in this systematic review are publicly available from the cited publications. The full study protocol and search strategy are archived and accessible at [https://doi.org/10.17605/OSF.IO/FV9PR]. No new primary data were generated.

## Supplementary Materials

The following supporting information can be downloaded at: https://www.mdpi.com/article/10.3390/antiox15010009/s1, The full PubMed search strategy, including MeSH terms, keywords, and Boolean operators, is provided in Supplementary File S1. Ref [178] was cited in the Supplementary Materials.

## Abbreviations

The following abbreviations are used in this manuscript:

14,15-EpETE/17,18-EpETE	14,15-Epoxyeicosatetraenoic Acid/17,18-Epoxyeicosatetraenoic Acid
14-HDHA/17-HDHA	14-Hydroxydocosahexaenoic Acid/17-Hydroxydocosahexaenoic Acid
5-HETE/12-HETE/12-HEPE/15-HEPE	5-Hydroxyeicosatetraenoic Acid/12-Hydroxyeicosatetraenoic Acid/12-Hydroxyeicosapentaenoic Acid/15-Hydroxyeicosapentaenoic Acid
5-oxo-ETE	5-Oxo-Eicosatetraenoic Acid
AA	Arachidonic Acid
AC	Acne Comedonica
AD/AD-lika	Atopic dermatitis/Atopic dermatitis like
ALA	α-Linolenic Acid
ALX/FPR2	Lipoxin A4 Receptor/Formyl Peptide Receptor 2
AP	Acne Papulopustulosa
AT/ATL	Aspirin triggered/Aspirin triggered lipoxin
BALB/c	Bagg Albino Laboratory-bred Mouse Strain c
BLT1	Leukotriene B4 Receptor 1
BSA	Body Surface Area
CBMC	Cord blood mononuclear cell
CCL2	C-C motif chemokine ligand 2
CD80/86	Co-stimulatory molecules expressed on antigen-presenting cells
CI	Confidence Interval
COX/COX-2/acetyl-COX-2	Cyclooxygenase/Cyclooxygenase-2/aspirin-acetylated cyclooxygenase-2
CRP	C-Reactive Protein
CTLA-4	Cytotoxic T-lymphocyte-associated protein 4
CYP-450	Cytochrome P450 Enzyme System
CXCL10	C-X-C motif chemokine ligand 10
DGLA	Dihomo-γ-Linolenic Acid
DHA	Docosahexaenoic Acid
DHA-M2	Docosahexaenoic acid–induced M2 macrophages
DHET	Dihydroxyeicosatrienoic Acid
DLQI	Dermatology Life Quality Index
DNCB	2,4-dinitrochlorobenzene
DNFB	2,4-dinitrofluorobenzene
DPA	Docosapentaenoic Acid
EASI	Eczema Area and Severity Index
ECM	Extracellular Matrix
ELOVL	Elongation of Very Long Chain Fatty Acids Protein (family)
EPA/EPA-E	Eicosapentaenoic Acid/eicosapentaenoic acid ethyl ester
ERK1/2	Extracellular Signal–Regulated Kinases 1/2
FAs	Fatty acids
FABP	Fatty Acid–Binding Protein
FADS2	Fatty acid desaturase 2
FDLQI	Family Dermatology Life Quality Index
FFA4R/GPR120	Free Fatty Acid Receptor 4
FK506	Tacrolimus
FMT	Fecal Microbiota Transplantation
GLA	γ-linolenic acid
GPCR	G Protein–Coupled Receptor
HDL	High-density lipoprotein
HETE	Hydroxyeicosatetraenoic Acid
HEPE	Hydroxy-eicosapentaenoic Acid
HMGB	High-Mobility Group Box protein
HpEPE	Hydroperoxy-eicosapentaenoic Acid
HpETE	Hydroxyperoxy-eicosatetraenoic Acid
HR-AD	Hairless atopic dermatitis
HRO	Herring Roe Oil
HS	Hidradenitis Suppurativa
IgE	Immunoglobulin E
IL	Interleukin
IRI/I–R	Ischemia–Reperfusion Injury
ISAAC	International Study of Asthma and Allergies in Childhood
IV	Intravenous
IMQ	Imiquimod
KO	Knockout
LCE3B–C	Late Cornified Envelope Genes 3B and 3C
LC-PUFA	Long-chain polyunsaturated fatty acid
LN	Lymph node
LOX/5-LOX/12-LOX/15-LOX	Lipoxygenase/5- Lipoxygenase/12- Lipoxygenase/15- Lipoxygenase
LPL	Lipoprotein Lipase
LPS	Lipopolysaccharide
LT/LTA_4/5_/LTE_4/5_	Leukotriene A_4/5_—E_4/5_
LXA4/LXA5	Lipoxin A4/A5
M2	Alternatively Activated Macrophage Phenotype
MAPK	Mitogen-Activated Protein Kinase
MaR1/MaR2	Maresin-1/Maresin 2
MCT	Medium-chain triglyceride
MCTR	Maresin Conjugates in Tissue Regeneration
MD	Mean difference
MR	Mendelian Randomization
MUFA	Monounsaturated fatty acid
NF-κB	Nuclear Factor kappa-light-chain-enhancer of activated B cells
NHDF	Normal Human Dermal Fibroblasts
NHEK	Normal Human Epidermal Keratinocytes
NK	Natural Killer Cells
NRF2	Nuclear Factor Erythroid 2–Related Factor 2
NSAID	Non-Steroidal Anti-Inflammatory Drug
PASI	Psoriasis Area and Severity Index
PAF	Platelet-activating factor
PBMC	peripheral blood mononuclear cell
PG/PGE_2_/PGE_3_/PGH_2_/PGG_2_	Prostaglandin/E2/E3/H2/G2
PGI2	Prostacyclin
PSO	Psoriasis
PUFA	Polyunsaturated Fatty Acid
PD1	Protectin D1
PDX	Protectin DX
PLA_2_/PLA2G4E/sPLA_2_	Phospholipase A_2_/Phospholipase A2 Group IVE/Secretory Phospholipase A2
PPAR	Peroxisome Proliferator–Activated Receptor
PRS	Polygenic risk score
RCT	Randomized Controlled Trial
QoL	Quality of life
RvD1-RvD6	Resolvin D1-D6
RvE/RvE1–RvE4	Resolvin E-series/E1/E2/E3/E4
S100A12	S100 Calcium-Binding Protein A12
SCORAD	SCORing Atopic Dermatitis
SFA	Saturated fatty acid
SNP	Single-nucleotide polymorphism
SPMs	Specialized Pro-Resolving Mediators
SSS	Symptom Severity Score
STAT1	Signal Transducer and Activator of Transcription 1
TCM	Central Memory T cells
TCS	Topical corticosteroid
TEWL	Transepidermal Water Loss
TG	Trigliceride
TGF-β	Transforming growth factor-β
Th/Th_2_/Th_17_	Helper T Cell/Type 2 Helper T Cell/Type 17 Helper T Cell
TN	Naïve T cells
TNF-α	Tumor Necrosis Factor α
T_reg_	Regulatory T cell
TSLP	Thymic stromal lymphopoietin
TXA_2_/TXB_2_	Thromboxane A_2_/B_2_
UFA	Unsaturated Fatty Acid
UV	Ultraviolet
VDR	Vitamin D Receptor
WT	Wild-Type
ω-3	Omega-3 Fatty Acids
ω-6	Omega-6 Fatty Acids
