# Supplementary material for: Specialized Pro-Resolving Lipid Mediators and Dietary Omega-3/6 Fatty Acids in Selected Inflammatory Skin Diseases: A Systematic Review
Source: Antioxidants (Basel). 2025 Dec 21;15(1):9. doi: 10.3390/antiox15010009 (PMC12837189; doi:10.3390/antiox15010009)
Supplement: Supplementary file 1 [file antioxidants-15-00009-s001.zip › Search strategy - supplementary material File S1.pdf]

A systematic literature search was conducted in **PubMed** using a combination of MeSH terms to identify studies on inflammatory skin diseases and specialized pro-resolving mediators (SPMs) or their fatty acid precursors. The search strategy was as follows:

("Psoriasis"[MeSH Terms] OR

"Dermatitis, Atopic"[MeSH Terms] OR

"Acne Vulgaris"[MeSH Terms] OR

"Hidradenitis Suppurativa"[MeSH Terms])

AND

("Lipoxins"[MeSH Terms] OR

"Docosahexaenoic Acids"[MeSH Terms] OR

"Eicosapentaenoic Acid"[MeSH Terms] OR

"Fatty Acids, Omega-3"[MeSH Terms] OR

"Fatty Acids, Omega-6"[MeSH Terms])

Boolean operators **AND** / **OR** were used to combine disease terms with mediator and fatty acid terms.

Only English-language articles were included.

No restrictions were applied regarding publication date.

Duplicate records were removed prior to screening, and titles, abstracts, and full texts were independently reviewed by two authors according to predefined inclusion/exclusion criteria.
